# Genetically defined elevated homocysteine levels do not result in widespread changes of DNA methylation in leukocytes

**DOI:** 10.1371/journal.pone.0182472

**Published:** 2017-10-30

**Authors:** Pooja R. Mandaviya, Roby Joehanes, Dylan Aïssi, Brigitte Kühnel, Riccardo E. Marioni, Vinh Truong, Lisette Stolk, Marian Beekman, Marc Jan Bonder, Lude Franke, Christian Gieger, Tianxiao Huan, M. Arfan Ikram, Sonja Kunze, Liming Liang, Jan Lindemans, Chunyu Liu, Allan F. McRae, Michael M. Mendelson, Martina Müller-Nurasyid, Annette Peters, P. Eline Slagboom, John M. Starr, David-Alexandre Trégouët, André G. Uitterlinden, Marleen M. J. van Greevenbroek, Diana van Heemst, Maarten van Iterson, Philip S. Wells, Chen Yao, Ian J. Deary, France Gagnon, Bastiaan T. Heijmans, Daniel Levy, Pierre-Emmanuel Morange, Melanie Waldenberger, Sandra G. Heil, Joyce B. J. van Meurs, on behalf of The CHARGE Consortium Epigenetics group and BIOS Consortium

**Affiliations:** 1 Department of Clinical Chemistry, Erasmus University Medical Center, Rotterdam, The Netherlands; 2 Department of Internal Medicine, Erasmus University Medical Center, Rotterdam, The Netherlands; 3 Institute for Aging Research, Hebrew SeniorLife, Harvard Medical School, Boston, MA, United States of America; 4 Sorbonne Universités, UPMC Univ. Paris 06, INSERM, UMR_S 1166, Team Genomics & Pathophysiology of Cardiovascular Diseases, Paris, France; 5 ICAN Institute for Cardiometabolism and Nutrition, Paris, France; 6 Research Unit of Molecular Epidemiology, Helmholtz Zentrum München—German Research Center for Environmental Health, Neuherberg, Germany; 7 Institute of Epidemiology II, Helmholtz Zentrum München—German Research Center for Environmental Health, Neuherberg, Germany; 8 Queensland Brain Institute, The University of Queensland, Brisbane, Australia; 9 Centre for Cognitive Ageing and Cognitive Epidemiology, University of Edinburgh, Edinburgh, United Kingdom; 10 Medical Genetics Section, Centre for Genomic and Experimental Medicine, Institute of Genetics and Molecular Medicine, University of Edinburgh, Edinburgh, United Kingdom; 11 Division of Epidemiology, Dalla Lana School of Public Health, University of Toronto, Toronto, Canada; 12 Molecular Epidemiology Section, Department of Medical Statistics and Bioinformatics, Leiden University Medical Center, Leiden, The Netherlands; 13 Department of Genetics, University Medical Center Groningen, Groningen, The Netherlands; 14 Framingham Heart Study, Framingham, MA, United States of America; 15 The Population Studies Branch, National Heart, Lung, and Blood Institute of the National Institutes of Health, Bethesda, MD, United States of America; 16 Department of Epidemiology, Erasmus University Medical Center, Rotterdam, The Netherlands; 17 Harvard T.H. Chan School of Public Health, Boston, MA, United States of America; 18 Department of Cardiology, Boston Children's Hospital, Boston, MA, United States of America; 19 DZHK (German Centre for Cardiovascular Research), partner site Munich Heart Alliance, Munich, Germany; 20 Institute of Genetic Epidemiology, Helmholtz Zentrum München—German Research Center for Environmental Health, Neuherberg, Germany; 21 Department of Medicine I, University Hospital Munich, Campus Grosshadern, Ludwig-Maximilians-University, Munich, Germany; 22 Department of Internal Medicine and School for Cardiovascular Diseases (CARIM), Maastricht University Medical Center, Maastricht, The Netherlands; 23 Department of Gerontology and Geriatrics Section, Leiden University Medical Center, Leiden, The Netherlands; 24 Department of Medicine, University of Ottawa, and the Ottawa Hospital Research Institute, Ottawa, Canada; 25 Department of Psychology, University of Edinburgh, Edinburgh, United Kingdom; 26 Laboratory of Haematology, La Timone Hospital, Marseille, France; 27 Institut National pour la Santé et la Recherche Médicale (INSERM), UMR_S 1062, Inra UMR_1260, Aix-Marseille Université, Marseille, France; Universitatsklinikum Hamburg-Eppendorf, GERMANY

## Abstract

**Background:**

DNA methylation is affected by the activities of the key enzymes and intermediate metabolites of the one-carbon pathway, one of which involves homocysteine. We investigated the effect of the well-known genetic variant associated with mildly elevated homocysteine: *MTHFR* 677C>T independently and in combination with other homocysteine-associated variants, on genome-wide leukocyte DNA-methylation.

**Methods:**

Methylation levels were assessed using Illumina 450k arrays on 9,894 individuals of European ancestry from 12 cohort studies. Linear-mixed-models were used to study the association of additive *MTHFR* 677C>T and genetic-risk score (GRS) based on 18 homocysteine-associated SNPs, with genome-wide methylation.

**Results:**

Meta-analysis revealed that the *MTHFR* 677C>T variant was associated with 35 CpG sites in *cis*, and the GRS showed association with 113 CpG sites near the homocysteine-associated variants. Genome-wide analysis revealed that the MTHFR 677C>T variant was associated with 1 *trans*-CpG (nearest gene *ZNF184*), while the GRS model showed association with 5 significant *trans*-CpGs annotated to nearest genes *PTF1A*, *MRPL55*, *CTDSP2*, *CRYM* and *FKBP5*.

**Conclusions:**

Our results do not show widespread changes in DNA-methylation across the genome, and therefore do not support the hypothesis that mildly elevated homocysteine is associated with widespread methylation changes in leukocytes.

## Introduction

DNA methylation, an important epigenetic mechanism has gained interest in the field of cancer and aging over the last decade [1, 2]. DNA methylation is affected by the activities of the key enzymes and intermediate metabolites of the one-carbon pathway, one of which involves homocysteine (Hcy).

Our aim was to investigate the role of genetically defined Hcy levels on genome-wide DNA methylation. A number of earlier studies have reported a link between Hcy and DNA methylation [3]. In these studies, DNA methylation was quantified as a global measure, that represents the total methyl cytosine content of the DNA. In animal models, both diet- and genetically- induced elevated Hcy have been related to altered global methylation patterns in tissues of aorta, brain, liver and colon. In human subjects, global DNA methylation in blood was not consistently altered with elevated Hcy. The relationship between Hcy and methylation can be subject to substantial bias, given the strong relationship between several lifestyle factors, diseases and Hcy. A way to circumvent this bias is to use genetic factors determining Hcy concentrations as an instrument to study the relationship between Hcy and methylation. The use of genetically defined elevated Hcy eliminate the effects that are possibly caused by measurement errors, confounding and reverse causality. One of the most consistent genetic variants causing elevated Hcy is the *MTHFR* 677C>T (rs1801133), which explains 5.3% variance in Hcy [4]. Furthermore, we recently published 18 variants including *MTHFR* 677C>T to be robustly associated with Hcy [5]. The Genetic Risk Score (GRS) of these 18 Hcy-associated variants explained 5.9% variance in Hcy [5]. In the current study, we used *MTHFR* 677C>T independently and the combined weighted GRS of these 18 variants, to test whether genetically defined elevated Hcy concentrations are associated with DNA methylation changes in blood cells.

A number of studies [3] have examined the relationship between the *MTHFR* 677C>T variant and global DNA methylation in humans. In 5 studies, individuals with the *MTHFR* 677TT genotype were compared to those with the *MTHFR* 677CC genotype [6–10]. Lower global methylation in blood cells was observed in two studies [6, 7]. The remaining three studies showed no association in the lymphocyte or colonic tissue. All published studies until now had small sample sizes of less than 200. To the best of our knowledge, associations of genetically defined Hcy with site-specific CpG methylation on a genome-wide scale have not been done up to now. In order to investigate this, we analyzed DNA methylation data measured with the Infinium Illumina 450k arrays, in a large meta-analysis of 9,894 individuals comprising 12 cohorts. We hypothesize that genetically defined elevated Hcy is associated with altered DNA methylation.

## Materials and methods

### Study population

All participants provided a written informed consent, and each study was approved at the relevant organizations by their respective ethics review committees [RS, Institutional review board (Medical Ethics Committee) of the Erasmus Medical Center; LLS, Ethical committee of the Leiden University Medical Center; LL, Ethics committee of the University Medical Centre Groningen; NTR, Central Ethics Committee on Research Involving Human Subjects of the VU University Medical Centre; CODAM, Medical Ethical Committee of the Maastricht University; MARTHA, “Departement santé de la direction générale de la recherche et de l'innovation du ministère” (Projects DC: 2008–880 & 09.576); EGCUT; Research Ethics Committee of the University of Tartu; F5L, Research ethics boards of the University of Toronto and the Ottawa Hospital Research Institute; FHS, IRB (institutional review board); KORA, Local Ethics Committee; LBC1921, Lothian Research Ethics Committee (Wave 1: LREC/1998/4/183); LBC1936, Multi-Centre Research Ethics Committee for Scotland (Wave 1: MREC/01/0/56), and the Lothian Research Ethics Committee (Wave 1: LREC/2003/2/29)].

The analyses comprised of large population with 9,894 participants from 12 cohorts of European ancestry. Most of the cohorts were part of either the Cohorts for Heart and Aging Research in Genomic Epidemiology (CHARGE) consortium [11] and/or Biobank-based Integrative Omics Studies (BIOS) consortium [12]. All participants provided a written informed consent for the DNA collection and its use for genetic analyses. Each study was approved at the relevant organizations by their respective ethics review committees. Cohort-specific characteristics are provided in the S1 Text and S1 Table.

### *MTHFR* 677C>T and homocysteine-associated SNPs

18 independent Hcy-associated SNPs from our GWAS meta-analysis [5], were selected to assess the relationship between mildly elevated Hcy concentrations and genome-wide DNA methylation. The genotypes of these SNPs were extracted from the genotyping data. Cohort-specific details of the quality control and the SNP imputation methods are provided in the S2 Table.

### DNA methylation assessment

Whole blood samples were collected from the participants for DNA extraction. The genomic DNA was bisulfite converted using the Zymo EZ-96 DNA-methylation kit (Zymo Research, Irvine, CA, USA). Methylation profiling was performed using the Infinium Illumina HumanMethylation 450k BeadChip arrays (Illumina Inc., San Diego, USA) according to the manufacturers’ protocol. Beta values from 0 to 1, which represent the percentage of methylation, were calculated from the extracted raw methylated (M) and unmethylated (U) probe intensities and a default alpha (α) of 100. This is defined by the formula of β = M/(M+U+α). Normalization was performed on these raw beta values using DASEN [13] or SWAN [14] methods. Poor quality probes were excluded based on the detection p-values mostly >0.01 in >5% of samples. Cohort-specific data preprocessing methods are provided in the Table 1. Global methylation levels per sample was calculated by the mean of all CpGs as well as mean according to CpG islands, shores, shelves or non-coding regions [15].

**Table 1:** Details of methylation 450k pre-processing. Quality control, normalization and association model.

		PROBES EXCLUSION				SAMPLE EXCLUSION	NORMALIZATION			ASSOCIATION MODEL			META-ANALYSIS
Sr. No.	Cohorts	Detection p-value criteria	Cross-reactive & polymorphic	XY	Final	Criteria (Method)	Background correction	Dye bias correction	Normalization method	WBC counts	Technical covariates	Additional adjustments	Probe exclusion
**CHARGE consortium**													Probes with SNPs at SBE & probes with improper binding [16], probes that were absent in 8 or more studies, *cis*-probes with <5 Mb distance from Hcy-SNPs
**1**	**RS-III**	>0.01 in >5% samples	Included	Excluded	463,456	Sample Call Rate >99%, poor bisulfite conversion, failed chromosome X & Y clustering	Yes	No	DASEN	Measured	Array, array position	No	
**2**	**LBC1921**	>0.01 in >5% samples	Included	Included	446,851	>0.01 det. p-value in >5% probes, poor bisulfite conversion	Yes	No	None	Measured	Array, array position, plate, hybridization date	No	
**3**	**LBC1936**	>0.01 in >5% samples	Included	Included	446,851	>0.01 det. p-value in >5% probes, poor bisulfite conversion	Yes	No	None	Measured	Array, array position, plate, hybridization date	No	
**4**	**KORA**	>0.01	Included	Included	441,487	>0.01 det. p-value in >20% probes	Yes	Yes	BMIQ	Imputed	Array, array position	No	
**5**	**FHS**	None	Included	Included	485,512	Mismatched sex, outliers based on principal components (PCs)	Yes	No	DASEN	Imputed	Array, array position, PCs	No	
**BIOS consortium**													
**6**	**RS**	>0.01 in >5% samples	Included	Excluded	419,937	Poor bisulfite conversion	Yes	No	DASEN	Imputed	Array, array position	No	
**7**	**LLS**	>0.01 in >5% samples	Included	Excluded	419,550	Poor bisulfite conversion	Yes	No	DASEN	Imputed	Array, array position	No	
**8**	**LLD**	>0.01 in >5% samples	Included	Excluded	420,591	Poor bisulfite conversion	Yes	No	DASEN	Imputed	Array, array position	No	
**9**	**NTR**	>0.01 in >5% samples	Included	Excluded	420,341	Poor bisulfite conversion	Yes	No	DASEN	Imputed	Array, array position	No	
**10**	**CODAM**	>0.01 in >5% samples	Included	Excluded	410,042	Poor bisulfite conversion	Yes	No	DASEN	Imputed	Array, array position	No	
**Other cohorts**													
**11**	**MARTHA**	>0.05 in >5% samples	Excluded	Included	388,120	Sample PCA	Yes	Yes	SWAN	Measured	Array, array position	No	
**12**	**F5L**	>0.05 in >5% samples	Excluded	Excluded	378,594	Sample PCA	Yes	Yes	SWAN	Imputed	Array, array position	Family structure	

### Statistical analysis

Two models were run independently by each participating study. Firstly, an additive model for *MTHFR* 677C>T alone was used to investigate its independent association with genome-wide DNA methylation in a linear manner. For the *MTHFR* 677C>T variant, genotypes were coded as CC = 0, CT = 1 and TT = 2 to study the effect in methylation per *MTHFR* 677T allele.

In the second analysis, a weighted Genetic Risk Scores (GRS) was constructed from all the 18 Hcy-associated variants to investigate their combined and additive effect on genome-wide DNA methylation. Weighted GRS were calculated on the basis of their effect sizes [5] and number of corresponding risk alleles. The product of the two was calculated for each SNP and then summed up for all SNPs. The GRS was calculated using the equation below, where N is the number of elevated Hcy causing risk alleles for each SNP (0, 1 or 2 per genotype).

$$\begin{array}{l} \mathrm{GRS}=0.1583\;x\;N\left(rs1801133:A\right)+\;0.0542\;x\;N\left(rs2275565:G\right)+\;0.0718\;x\;N\left(rs234709:C\right)+\\ 0.0435\;x\;N\left(rs4660306:T\right)+\;0.0453\;x\;N\left(rs1801222:A\right)+\;0.101\;x\;N\left(rs12134663:C\right)+\\ 0.0529\;x\;N\left(rs12780845:A\right)+\;0.056\;x\;N\left(rs2851391:T\right)+\;0.0449\;x\;N\left(rs9369898:A\right)+\\ 0.0422\;x\;N\left(rs838133:A\right)+\;0.0864\;x\;N\left(rs7422339:A\right)+\;0.1242\;x\;N\left(rs7130284:C\right)+\\ 0.0963\;x\;N\left(rs154657:A\right)+\;0.0597\;x\;N\left(rs548987:C\right)+\;0.0395\;x\;N\left(rs42648:G\right)+\\ 0.0512\;x\;N\left(rs2251468:C\right)+\;0.045\;x\;N\left(rs957140:G\right)+\;0.090\;x\;N\left(rs12921383:C\right) \end{array}$$ (1)

Both the analyses were based on linear mixed models of lme4 package in R. We also analyzed the effect of *MTHFR* 677C>T and GRS on global methylation levels, where we calculated the overall mean levels per individual as well as categorized the means as per CGI annotations [15]. The models were adjusted for technical covariates and biological covariates like age, sex and differential white blood cell (WBC) counts (see S1 Table for details about covariates for each cohort). The technical covariates were cohort-specific and treated as random effects. WBC counts were either used as measured counts, or they were imputed based on the Houseman method as implemented in the minfi package [17], or the modified version of the Houseman method (Documentation and R script: https://github.com/mvaniterson/wbccPredictor) that uses partial least-squares [18] to handle multivariate responses and high-dimensional covariates and has been previously used [19]. This method from van Iterson used the R package pls [18] to fit the linear model based on the DNA methylation data, to predict the white blood cell composition as percentages that sum up to almost 100%. Age and gender were used as covariates.

### Meta-analysis

Summary statistics for the two models were obtained from each study. Because of the different probe exclusions in each cohort [Table 1], we removed probes that were present in ≤4 studies. We also excluded probes with SNPs at single base extension site, and probes with improper binding [12], leaving a total of 465,694 probes for the meta-analysis. Meta-analysis was performed using the fixed effect model in METAL [20], with the classical approach that uses effect size estimates and standard errors as input obtained from the individual study summary statistics for each CpG probe. The output of meta-analysis gave the combined effect size estimates, standard errors and p-values per probe. These p-values were corrected using the Benjamini Hochberg method of false discovery rate (FDR), where FDR <0.05 was considered statistically significant. For the MTHFR 677C>T model, positive effect sizes correspond to percentage increase in methylation per *MTHFR* 677T allele. For the GRS model, positive effect sizes correspond to percentage increase in methylation per unit increase in GRS. We also took into account heterogeneity of the meta-analysis by I^2^ which was calculated using METAL per probe and excluded significant probes if I^2^<40. We also calculated the genomic inflation factor (λ) [21] to estimate the inflation in test statistics that may be caused by population structure or other unknown confounding factors. This λ was estimated for the distribution of p-values using the median method, which is defined as the ratio of the observed median of the test statistic distribution and the expected median 0.455 [21, 22].

Genomic Regions Enrichment of Annotations Tool (GREAT) was used for annotating CpGs for nearby genes, that assigns a basal regulatory region to extend up to 5 kb upstream and 1 kb downstream from its transcription start site and a maximum extension distance up to 1 Mb [23], as defined by UCSC [24]. Furthermore, strength of the instrument or allele score was calculated using the F-statistics, using the tool, mRnd [25]. We took into account cohort heterogeneity I^2^ and excluded significant probes if I^2^<40.

### Identifying *cis*- and *trans*-CpG effects

We defined the CpGs as “*cis*” when the CpG was annotated within 1Mb upstream or downstream of the SNP. *Trans*-CpGs were defined as CpGs that were associated with the SNP, and were annotated >1Mb apart. We defined the CpGs in the GRS model the same way by accounting for the bp distance of the CpGs from each of the 18 SNPs. For the significant *trans*-CpGs that were 1-5Mb apart from any of the 18 SNPs, we performed a conditional analysis adjusting for that SNP to investigate whether they were *trans*-CpGs associated with Hcy GRS or long range *cis*-CpGs driven by the nearby SNPs that were part of the GRS. In the conditional analysis, if the bonferroni corrected p-values were no longer significant, we considered those trans-CpGs as long-range cis-CpGs. For the significant *trans*-CpGs that were >5Mb apart from any of the 18 SNPs, we looked up for their tested individual association with each of the 18 SNPs in our previous *trans*-CpG mapping analysis [12]. This is to see whether the association of these *trans*-CpGs was Hcy GRS driven or driven by a single SNP that was a part of GRS. In order to confirm these *trans*-CpG effects, we performed a similar conditional analysis by including the respective SNP as a covariate in the model. Both conditional analyses were performed on a subset of 3,786 samples from 6 cohorts, and the results were compared with the unconditional analysis in the same subset.

### H19/IGF locus

Three Differentially Methylated Regions (DMRs) of IGF2/H19 locus at chromosome 11 have been reported to be related with homocysteine [26, 27]. We identified seven CpGs on the 450k array that were underlying the three DMRs of this locus, for their association with MTHFR 677C>T variant or GRS. Bonferroni method was applied on these 7 CpGs to check for multiple testing.

### Enrichment of folate-associated CpGs

We further focused our analysis on the 443 previously identified CPGs, of which methylation in cord blood of newborns were associated with maternal plasma folate levels [28]. We compared the p-values of these 443 CpGs from the MTHFR 677>T and the GRS results, and compared them to the p-values of 100 random CpGs with 1000 permutations, to check for their significant enrichment, using the Fisher’s exact test.

## Results

### Population characteristics

The meta-analysis included 9,894 adults from 12 cohorts. Studies were population-based, except for the Cohort on Diabetes and Atherosclerosis Maastricht, MARseille THrombosis Association study and the French-Canadian family study, where individuals were selected based on mildly increased diabetes mellitus type 2 and cardiovascular risk factors, cases of venous thrombosis and probands with venous thromboembolism, respectively.

### Meta-analysis of MTHFR 677C>T and GRS model

**Fig 1:**
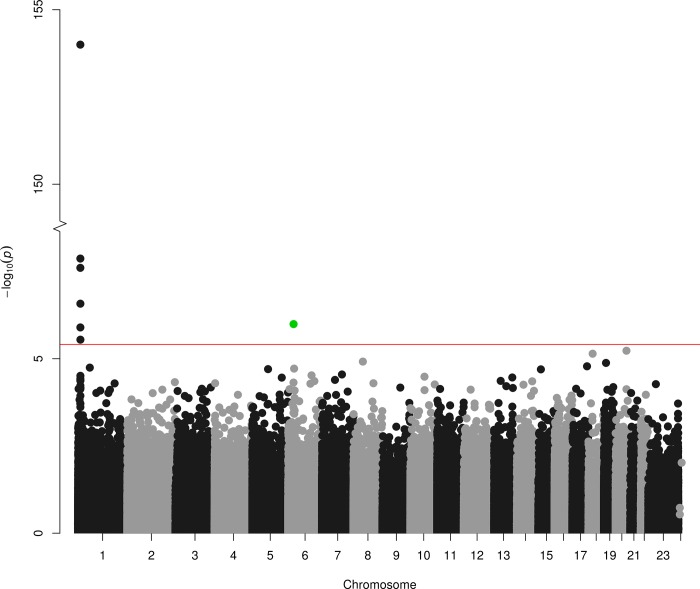
Manhattan plot. Association between MTHFR 677C>T (rs1801133) and genome-wide DNA methylation in 9,894 samples, with 35 *cis*-meQTLs at chromosome 1 (black/grey) and 1 *trans*-meQTL at chromosome 6 (green) with FDR<0.05.

**Fig 2:**
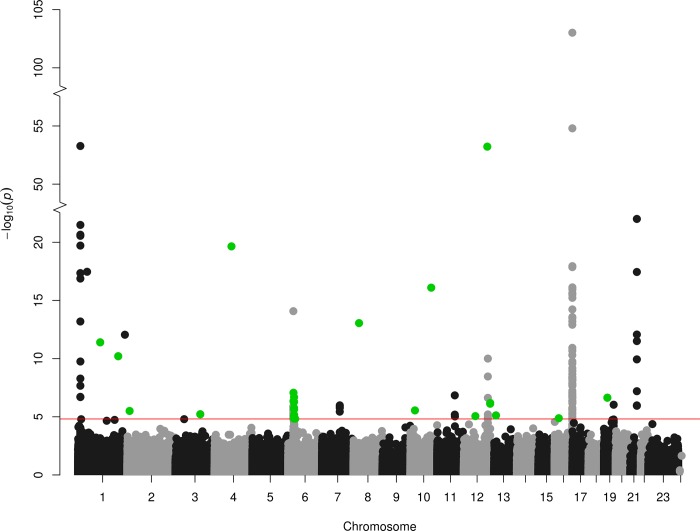
Manhattan plot. Association between GRS of 18 Hcy-associated SNPs and genome-wide DNA methylation in 9,894 samples, with 113 *cis*-meQTLs (black/grey) and 30 *trans*-meQTLs (green), at FDR<0.05.

The explained variance in Hcy by MTHFR 677C>T is 5.3% [4] and by GRS is 5.9% [5]. For a sample size of 9,894, the F-statistics of the additive MTHFR 677C>T and GRS was 554 and 621, respectively. Meta-analysis of 456,694 probes identified 35 *cis*- [S3 Table] and 1 *trans-* [S5 Table, Table 2] CpGs for the MTHFR model [Fig 1] and 113 *cis-* [S4 Table] and 30 *trans-* [Table a in S6 Table, Table a in S7 Table and S8 Table] CpGs for the GRS model [Fig 2]. The λ was 1.01 for the MTHFR 677C>T SNP and 0.92 for GRS [S1 Fig].

**Table 2:** Genome-wide *trans*-CpGs with FDR<0.05; associated with the *MTHFR* 677C>T model or Genetic Risk Score of 18 Hcy-associated variants.

*CpG*	*N*	*Beta*	*SE*	*P*	*FDR*	*I*^*2*^	*Beta*	*SE*	*P*	*Nearest Genes*	*Chr*	*Bp*
		***MTHFR* 677C>T model**					**LOOKUP in GRS model**					
cg05411165	9894	-0.005	0.001	1.02E-06	1.40E-02	0	-0.0095	0.0038	1.16E-02	*ZNF184* (-25414), *HIST1H2BL* (+309398)	6	27466334
		**GRS model**					**LOOKUP in *MTHFR* 677C>T model**					
cg12805629	6277	-0.018	0.004	3.15E-06	1.23E-02	0	-0.0023	0.0011	3.81E-02	*MRPL55* (+6698), *ARF1* (+19954)	2	11565653
cg08586216^+^	9894	-0.002	0.001	1.49E-05	4.89E-02	37.6	-0.0003	0.0001	4.17E-02	*TULP1* (-131681), *FKBP5* (+44391)	6	35612351
cg00620062	9894	0.004	0.001	2.83E-06	1.12E-02	0	0.0007	0.0003	3.36E-03	*PTF1A* (+6292)	10	23487775
cg00677455*	9334	-0.003	0.001	8.76E-06	3.09E-02	0	-0.0004	0.0002	3.63E-02	*CTDSP2* (-269)	12	58241039
cg01259782	6194	0.015	0.004	1.33E-05	4.52E-02	0	0.0021	0.0010	2.68E-02	*CRYM* (+454)	16	21313973

Beta: Regression coefficients

SE: Standard errors of the regression coefficients

FDR: False discovery rate adjusted P-value, threshold = 0.05

I^2^: Heterogeneity I^2^ parameter

*Promoter-associated

^+^Enhancer-associated, Enhancer annotation from Illumina 450k annotation

### *Cis*-CpGs

**Fig 3:**
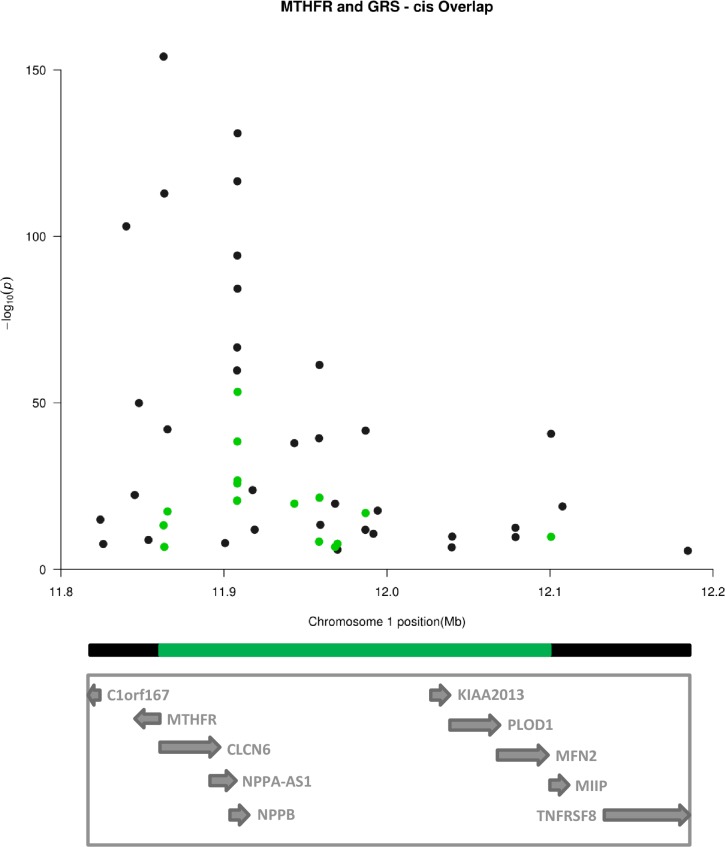
Regional manhattan plot (chr1: 11824095–12184574). 35 (black) and 16 (green) cis-meQTLs of the MTHFR 677C>T and GRS model respectively, in 9,894 samples. The overlap involved a small region of 238 kb (green rectangular line).

Meta-analysis on 465,694 CpGs of the *MTHFR* 677C>T variant showed association with 35 *cis*- CpGs on chromosome 1 with FDR<0.05 [Fig 1, S3 Table]. These *cis*-CpGs showed a range from 2.4% increase to 1.7% decrease in methylation per *MTHFR* 677T allele. The nearest genes associated with this *cis*-region included *MTHFR* itself, *AGTRAP*, *CLCN6*, *NPPA*, *NPPB*, *PLOD1*, *MFN2* and *TNFRSF8*. For the GRS model, we observed 113 *cis*-CpGs with FDR<0.05 [Fig 2, S4 Table]. Out of the 113, 16 *cis-*CpGs showed overlap with the MTHFR 677C>T analysis, which involved a smaller region of 238 Kb [Fig 3].

### *Trans*-CpGs

For the *MTHFR* 677C>T model, meta-analysis of 465,694 CpGs identified 1 significant *trans*-CpG which was located on chromosome 6 [Fig 1, S5 Table]. This *trans*-CpG (cg05411165) showed 1% decrease in methylation per MTHFR T allele. It was annotated near *ZNF184* (25414 bp upstream) and *HIST1H2BL* (309398 bp downstream). For the GRS model, we observed 30 significant *trans*-CpGs [Fig 2]. These *trans*-CpGs showed a range from 5.6% increase to 5.1% decrease in methylation per 0.1 unit increase in GRS. Of these 30 *trans*-CpGs, 23 were negatively associated with the GRS model. To assess overlap between two models, we evaluated association of the trans-CpG of the MTHFR 677 C>T model within the GRS model. This *trans*-CpG (cg05411165) showed a 10% decrease in methylation in the GRS model but was not FDR significant (raw p-value = 0.01).

Critical evaluation of the 30 *trans-*CpGs of GRS model demonstrated that 14 *trans-*CpGs were located in a large region of 3,08 Mb length within chromosome 6. The GRS model consists of 18 SNPs including a SNP on chromosome 6. The 14 trans CpG identified with the GRS model were at a distance between 1 and 5 Mb away from the Hcy-associated variant rs548987 of the *SLC17A3* gene at chromosome 6 [Table a in S6 Table].

#### Conditional analysis: Chromosome 6 region near rs548987

To investigate whether the 14 *trans*-CpGs on chromosome 6 near rs548987 were influenced by this variant, we performed conditional analysis on a subset of 3,786 samples from 6 cohorts. After correction of the model for rs548987 as a covariate, none of the 14 *trans*-CpGs were significant at a bonferroni threshold of 3.57E-03 [Table b in S6 Table].

#### Conditional analysis: Influence of SNPs within the GRS model

To further investigate the remaining 16 *trans-*CpGs from the 30, whether any of them were driven by a single variant, rather than the combined effect of the 18 homocysteine-associated variants, we checked the *trans*-CpG mapping analysis of the single SNPs using the BIOS dataset [12]. We observed that 7 of the 16 remaining *trans*-CpGs located >5 Mb from the Hcy-associated variants, were directly associated with either rs548987 SNP of *SLC17A3* gene at chromosome 6, or rs154657 SNP of *DPEP1* gene at chromosome 16 [Table a in S7 Table]. After correction for these 7 *trans*-CpGs by including the respective SNP as a covariate in the model, none of the 7 *trans*-CpGs remained significant at a bonferroni threshold of 7.14E-03 [Table b in S7 Table]. After correction for cis-effects of Hcy-associated SNPs in the GRS model, we identified a remaining list of 9 Hcy-associated *trans*-CpGs, 4 of which had substantial heterogeneity I^2^ [S8 Table].

### Overlapping *trans*-CpG between MTHFR and GRS models

When doing a lookup in the *MTHFR* 677C>T model for the finally identified 5 *trans-*CpGs, all of them showed similar direction of effect, but did not achieve genome-wide significance (Lowest raw p-value = 3.36E-03) [Table 2].

### *Trans-*CpGs affecting Gene expression

We evaluated whether methylation levels of the observed *trans*-CpG from the *MTHFR* 677C>T model and 5 *trans*-CpGs from the GRS model were associated with expression levels of the nearby genes, in the BIOS dataset [12]. None of the *trans*-CpGs was associated with mRNA expression differences of nearby genes.

### H19/IGF2 locus

We specifically focused on the IGF2-H19 region for differential methylation, since methylation at this locus has repeatedly been linked to the homocysteine metabolism in a number of studies [29–31]. S4 Fig shows the results of the whole IGF2-H19 region, and the 7 CpGs annotated to 3 DMRs of the IGF2/H19 gene that had previously been reported to be differentially methylated (DMR0, DMR2, H19-DMR3). Data from our 450k arrays contained 2 CpGs at DMR0, 4 CpGs at DMR2 and 1 CpG at H19-DMR3. None of them showed an association with MTHFR 677C>T or GRS with a Bonferroni cut off of 7.14E-03 [S9 Table, S4 Fig].

### Enrichment of previously found folate-associated CpGs

Next we focused on a set of 443 CpGs that were identified to be differentially methylated in children at birth according to the folate levels in the mothers [28]. We found a highly significant enrichment for significant p-values in the MTHFR model in the 443 CpGs as compared to a random set of other CpGs (>3 times enrichment of significant p-values, enrichment p = 0.0079). However, we did not find a significant enrichment for the GRS model.

### Global DNA methylation changes

In addition to genome-wide DNA methylation changes we analyzed the effect of *MTHFR* 677C>T and GRS models on overall mean methylation levels. There was no significant association between the *MTHFR* 677C>T or GRS on global methylation overall or mean methylation of CpG islands, shores, shelves or non-coding regions [Table 3] [15].

**Table 3:** Association of *MTHFR* 677C>T and Genetic Risk Score on mean global methylation levels.

*Methylation*	*N*	*I2*	*Beta*	*SE*	*P*
***MTHFR* 677C>T**					
GLOBAL	3,786	0.14	4.00E-06	1.50E-05	0.81
CGI	3,786	0.58	-1.90E-05	4.90E-05	0.70
SHE	3,786	0.59	3.70E-05	5.50E-05	0.50
SHO	3,786	0.14	-9.00E-06	4.80E-05	0.85
NC	3,786	0.61	2.70E-05	4.90E-05	0.57
**GRS**					
GLOBAL	3,786	0.67	-1.30E-05	5.60E-05	0.81
CGI	3,786	0.07	-1.84E-04	1.81E-04	0.31
SHE	3,786	0.00	2.29E-04	2.01E-04	0.25
SHO	3,786	0.52	-1.20E-04	1.76E-04	0.50
NC	3,786	0.07	1.94E-04	1.78E-04	0.28

Beta: Regression coefficients

SE: Standard errors of the regression coefficients

I^2^: Heterogeneity I^2^ parameter

CGI = CpG Islands, SHE = CpG Shelves, SHO = CpG Shores, NC = CpGs at Non-Coding regions

## Discussion

This is the first large-scale study to investigate the effect of Hcy-associated SNPs on genome-wide DNA methylation using the Illumina 450k arrays in 9,894 individuals. The results showed no widespread *trans*-effects of the *MTHFR* 677C>T SNP on DNA methylation, apart from 1 *trans*-CpG at chromosome 6. The GRS model showed 5 *trans*-CpGs, after carefully examining the direct effects of individual SNPs with conditional analyses.

In this current study, we used *MTHFR* 677C>T independently and the combined weighted GRS of the 18 Hcy-associated variants [5], to test whether mildly elevated Hcy concentrations induce DNA methylation changes in blood cells. Our goal was to investigate genetically defined elevated Hcy on genome-wide DNA methylation. The use of genetic variants is less sensitive to confounding and bias as compared to classical epidemiological studies [32].

We calculated the strength of our exposure variables: *MTHFR* 677C>T and GRS using the F-statistics. For a strong exposure, the value of the F-statistics is expected to be greater than 10 [33]. With our large sample size (n = 9,894) and proportion of variance explained being 5.3 to 5.9%, the F-statistics was 554 and 621 respectively, indicating very high strength and enough power of our analysis. However, we did not observe widespread *trans*-effects despite of having strong additive *MTHFR* 677C>T and GRS.

We observed a single *trans-*CpG for the *MTHFR* 677C>T variant. This CpG is located near *ZNF184* and *HIST1H2BL*. Both these genes are thought to play a role in transcriptional regulation. For the GRS, we found 30 *trans-*CpGs associated, 14 of which are spread over a region of 3,08 Mb at chromosome 6. These CpGs were annotated to genes that included *ZNF322* and *HIST1H2BJ*, *HLA-J*, *HLA-A*, *HLA-G*, but also the proximal region of *ZFP57* gene, which was previously identified as a folate-sensitive region in a genome-wide methylation study of 23 women [34]. However, when we performed a conditional analysis on these 14 CpGs in this region, by adjusting for the nearby variant rs548987 of the *SLC17A3* gene, the effect sizes significantly attenuated and the nominal p-values were no more significant. The results indicate that this region was influenced by the rs548987 SNP of *SLC17A3* gene and was not Hcy-associated.

We finally observed 5 *trans*-CpGs associated to genetically defined Hcy using the GRS, after carefully examining the direct effects of individual SNPs with conditional analyses and discarding CpGs that showed substantial cohort heterogeneity I^2^. A total of 3 CpGs showed hypomethylation, one of which was annotated to the *FKBP5* gene. *FKBP5* encodes for the FK506-binding protein 51 (FKBP51) whose expression has recently been shown to decrease DNMT1 activity and thereby decreasing global methylation [35].

Furthermore, when looking at our methylation-expression results [12], none of the 5 *trans*-CpGs was associated with mRNA expression differences of nearby genes. The possible explanation for these negative findings could be that these CpG sites might have an effect further away on trans-genes. Conversely, it has been shown that the methylation-expression correlation in cis are not best predicted using the CpG position alone, but by using specific chromatin marks [36]. Furthermore, it could also be that these correlations are specific to other tissues, but not in blood.

We observed that the IGF2-H19 locus did not show association with methylation according to genetically defined elevated homocysteine. This is in contrast to the previous findings in mice, where tissue-specific changes in H19 DMR methylation were found in liver, brain and aorta, and increased expression of H19 was found in aorta [26, 27]. Similar to what we found, the H19-DMR3 between CBS deficient patients and controls also did not show a significant difference, in a previous study [29]. Our results show that this imprinted locus is not deregulated by long-term genetically defined mildly elevated homocysteine. However, previously it was reported that MTHFR 677C>T variant shows changes in DNA methylation in peripheral blood mononuclear cells, only through an interaction with folate [7]. Hence, further studies are needed to study the effect of MTHFR 677C>T variant in the presence of blood folate levels.

We did not see widespread methylation changes associated to mildly elevated plasma Hcy concentrations. This result is not in line with a number of earlier reports, which have shown global methylation changes in association with the *MTHFR* 677C>T variant and Hcy concentrations [3]. Previous two studies on this topic have shown contradictory results. There has been reports that showed a lower circulating global methylation level in individuals with the *MTHFR* 677TT genotype [6]^,^[7]. However, there are also a few negative studies that showed no relation between the *MTHFR* 677TT genotype and global methylation levels [8–10]. All these studies had modest sample sizes (upto 300 individuals were studied), and measured methylation on a global level using the LINE-1 assay, which measures a repetitive sequence, of which the function is unknown. In contrast, we here studied a genome wide site-specific analysis focused on functional regions of the genome [15]. We here show convincing evidence that there is no association between the *MTHFR* 677C>T or GRS on overall methylation levels, nor is there a relationship between methylation of CpG islands, shores, shelves or non-coding regions separately, which supports the previous null associations.

Furthermore, in order to test for causal effect in a mendelian randomization study, an instrument, which is in our case MTHFR 677C>T or GRS, should satisfy the 3 basic assumptions [37, 38]. One, the instrument should be associated with the exposure, which is in our case Hcy. Two, the instrument should not affect the outcome, which is in our case DNA methylation, except through the exposure Hcy. Three, the instrument should not be associated with any confounder of the exposure-outcome association. Although assumptions one and two are satisfied in our case [5], the GRS model might violate assumption three [37, 38].

The GRS model contains a few SNPs which are, in addition to the association with Hcy, also associated with other traits. For example, the variants near to HNF1A gene have been associated with a number of other traits [39–44]. This could also be the reason why the results of the MTHFR 677C>T and GRS models are quite different. Nevertheless, the MTHFR 677C>T variant explains most of the variation in Hcy, as compared to the other variants in the GRS model and is therefore a strong instrument to examine the effect of deregulation of the one-carbon metabolism on methylation.

The relationship between *MTHFR* 677C>T and DNA methylation is modified by folate levels. Only in individuals with low folate status, the effect of the *MTHFR* 677TT genotype is seen [7]. Unfortunately, we were unable to study this interaction, since folate levels were not available in our study. Another prerequisite to be able to perform MR is that the relationship between Hcy and methylation is known. The relationship between Hcy and DNA methylation is only known in studies until now where methylation is measured at a global level. Therefore, the estimation of the causal effect could not be done. Unfortunately, we also did not have Hcy data available in all cohorts of this study, and therefore were unable to perform a full mendelian randomization study. We rather focused on the association of genetically defined elevated Hcy levels with DNA methylation.

We did not find widespread differences in methylation related to genetically defined homocysteine levels. The association was not observed in global methylation levels nor in widespread CpGs including the previously known H19/IGF2 locus. There are a number of possible explanations for this finding. First, it is known that the relationship between *MTHFR* 677C>T and DNA methylation is modified by folate levels, as described above. The effect of *MTHFR* 677C>T is seen in individuals with low folate status [7], which could have masked possible relationships between the MHTFR variant and methylation. A second possible explanation for the relative low number of identified CpGs, is that we have studied the wrong tissue. Most methylation measures are conducted in blood leukocytes as this tissue is readily available. However, the causal effect of Hcy could be specific to other tissues like liver, heart and brain. Therefore, the possible effect of mildly elevated Hcy on such specific tissues cannot be excluded. Third, there is little variation in the one-carbon metabolism in the normal population. This metabolism is pivotal to cell survival and function and therefore tidily regulated. It could be that there is a correlation between homocysteine and more pronounced effects on methylation when homocysteine levels are more extreme.

## Conclusions

We observed 1 *trans*-CpG (nearest genes *ZNF184* and *HIST1H2BL*) on chromosome 6 associated with the *MTHFR* 677C>T variant. The GRS model showed 5 significant *trans*-CpGs, which do not overlap with the MTHFR trans-CpG. In conclusion, our results do not show widespread statistically significant *trans*-effects of MTHFR and GRS models, and therefore do not support the hypothesis that genetically defined mildly elevated Hcy concentrations are associated with widespread methylation changes in leukocytes. More studies with measured Hcy concentrations are needed to confirm this.

## Supporting information

S1 Fig Quantile-quantile plots. Association of (a) MTHFR 677C>T and (b) Genetic risk score of all the 18 Hcy-associated variants with genome-wide DNA methylation.
(PDF)

S2 Fig Forest plot. *Trans*-meQTL of the MTHFR 677C>T model across 12 cohorts.
(PDF)

S3 Fig Forest plots. *Trans*-meQTLs of the Genetic risk score model across 12 cohorts.
(PDF)

S4 Fig Regional manhattan plots. (a) MTHFR 677 C>T variant or (b) Genetic risk score associated 3 DMRs of IGF2/H19 genes containing 7 CpGs (green) from the 450k data; DMR0 “Chr.11:2,170,380–2,170,517” with 2 CpGs, DMR2 “Chr.11:2,154,113–2,154,414” with 4 CpGs and H19-DMR3 “Chr.11:2,021,072–2,021,273” with 1 CpG.
(PDF)

S1 Table Cohort characteristic. (PDF)

S2 Table Details of genotyping methods. Quality control of SNPs and imputation.
(PDF)

S3 Table 35 *cis*-meQTLs with FDR<0.05 that are associated with MTHFR 677C>T variant (rs1801133) in a samples size of 9,894. The CpGs are sorted in base pairs.
(PDF)

S4 Table 113 *cis*-meQTLs with FDR<0.05 that are associated with Genetic Risk Score of 18 Hcy-associated variants in a sample size of 9,894. The CpGs are sorted in chromosomes and base pairs.
(PDF)

S5 Table *Trans*-meQTLs with FDR<0.05 that are associated with MTHFR 677C>T variant (rs1801133) in a sample size of 9,894. (PDF)

S6 Table **(a) 14 *trans*-meQTLs of chromosome 6 with FDR<0.05 that are associated with Genetic Risk Score of 18 Hcy-associated variants in a sample size of 9,894, and were 1Mb-5Mb away from the SNP rs548987 of SLC17A3 gene. (b) Conditional analysis for the 14 *trans*-meQTLs of chromosome 6 with adjustment for the SNP rs548987 of SLC17A3 gene in a subset of 3,786.** The CpGs are sorted in base pairs.
(PDF)

S7 Table (a) 7 genome-wide *trans*-meQTLs with FDR<0.05 that are associated with Genetic Risk Score of 18 Hcy-associated variants in a sample size of 9,894 and were a direct *trans*-meQTL of either SNP rs548987 of SLC17A3 gene or rs154657 SNP of DPEP1 gene. (b) Conditional analysis for the 7 genome-wide *trans*-meQTLs with adjustment for their respective SNP rs548987 of SLC17A3 gene at chromosome 6 or rs154657 SNP of DPEP1 gene at chromosomes 16 in a subset of 3,786.
(PDF)

S8 Table 9 genome-wide *trans*-meQTLs with FDR<0.05 that are associated with Genetic Risk Score of 18 Hcy-associated variants in a sample size of 9,894. The CpGs are sorted in chromosomes and base pairs.
(PDF)

S9 Table (a) MTHFR 677C>T variant associated DMPs at the 3 IGF2/H19 DMR regions at chromosome 11. (b) GRS associated DMPs at the 3 IGF2/H19 DMR regions at chromosome 11.
(PDF)

S1 Text Description of cohorts. (PDF)

S1 File Membership list of the BIOS consortium. (PDF)

S2 File Membership list of the CHARGE consortium. (PDF)
