# Supplementary material for: Genetically defined elevated homocysteine levels do not result in widespread changes of DNA methylation in leukocytes
Source: PLoS One. 2017 Oct 30;12(10):e0182472. doi: 10.1371/journal.pone.0182472 (PMC5662081; doi:10.1371/journal.pone.0182472)
Supplement: S1 Fig — Association of (a) MTHFR 677C>T and (b) Genetic risk score of all the 18 Hcy-associated variants with genome-wide DNA methylation. (PDF) [file pone.0182472.s001.pdf]

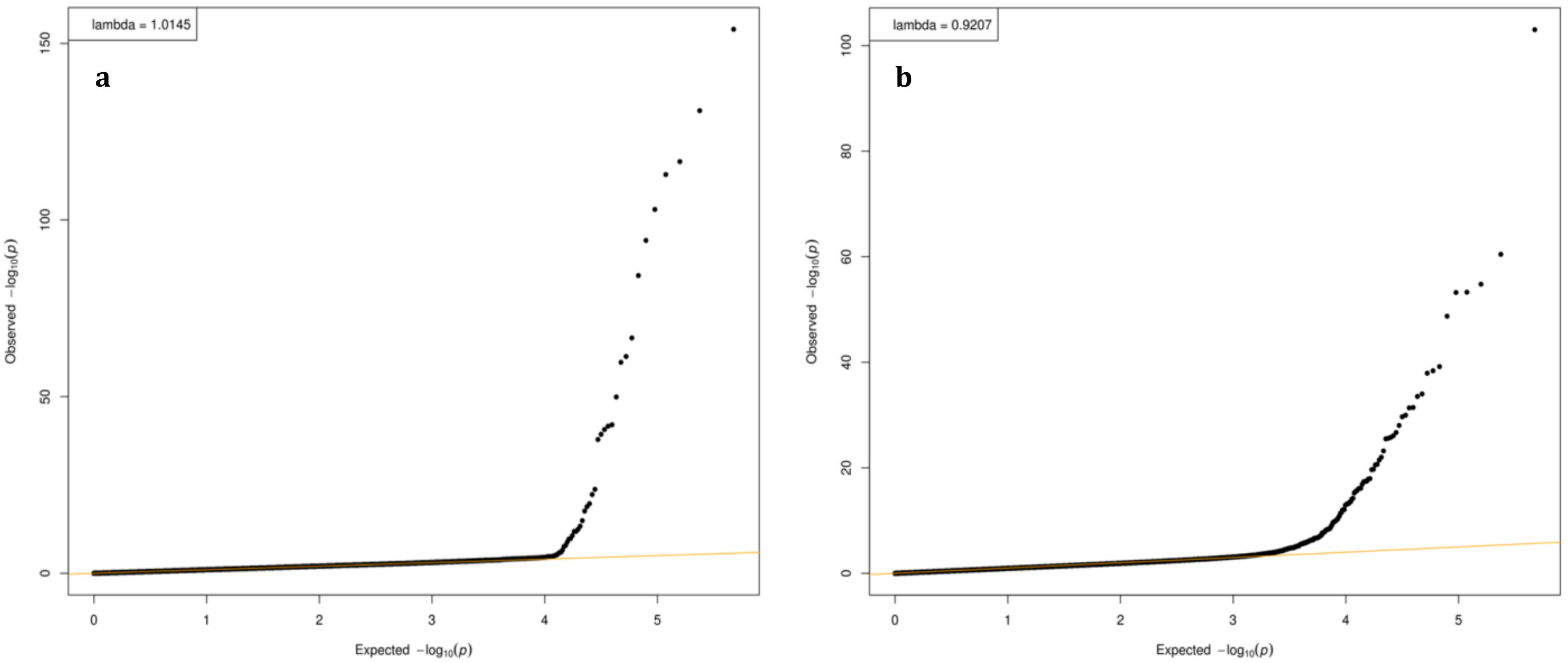

S1 (a and b) Figs. Quantile-Quantile plots. Association of (a) MTHFR 677C>T and (b) Genetic risk score of all the 18 Hcy-associated variants with genome-wide DNA methylation.
