# Supplementary figures and images for: Genetically defined elevated homocysteine levels do not result in widespread changes of DNA methylation in leukocytes
Source: PLoS One. 2017 Oct 30;12(10):e0182472. doi: 10.1371/journal.pone.0182472 (PMC5662081; doi:10.1371/journal.pone.0182472)

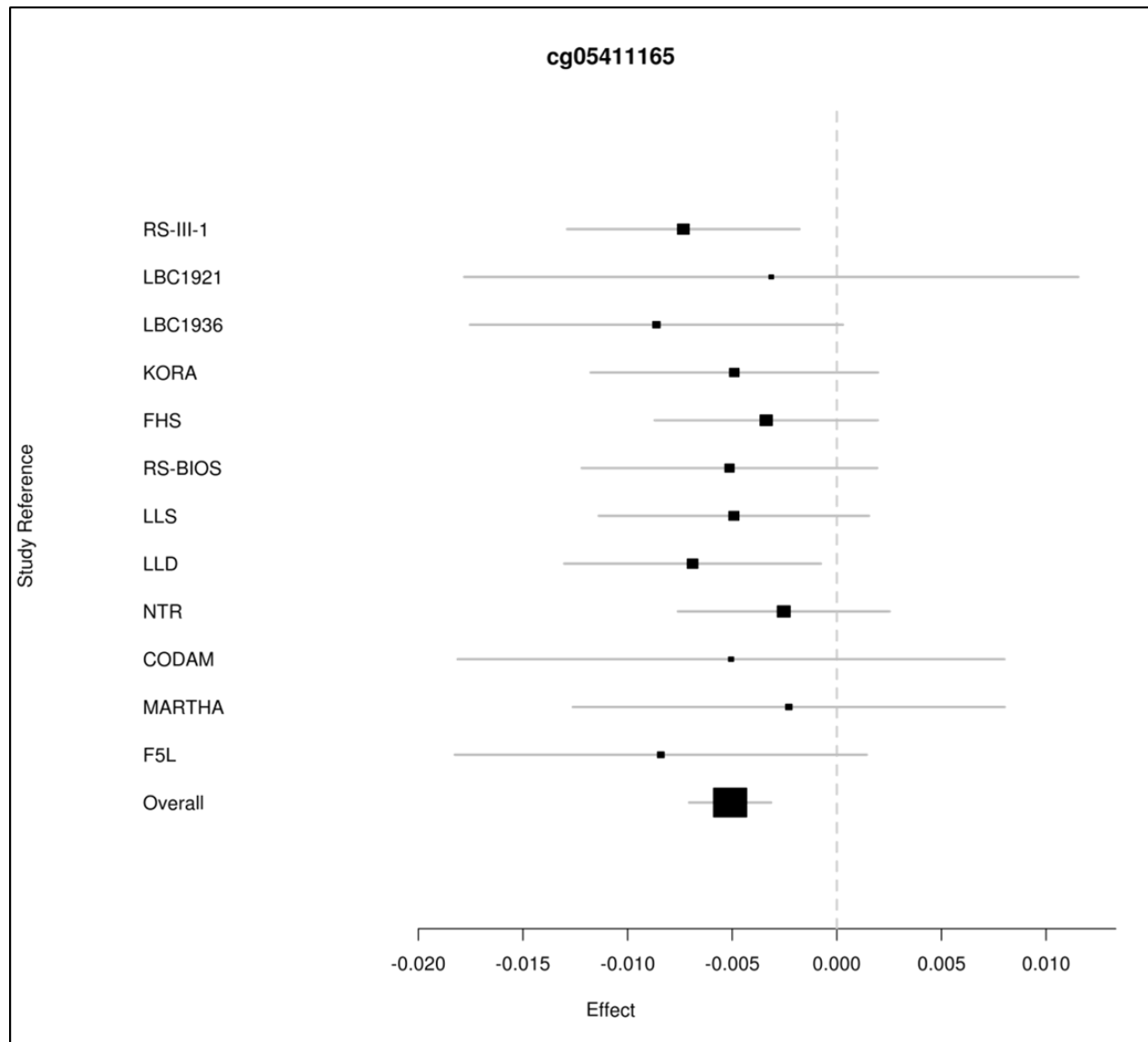

**S2 Fig. Forest plot.** *Trans*-meQTL of the MTHFR 677C>T model across 12 cohorts.

Supplement: S2 Fig — Trans-meQTL of the MTHFR 677C>T model across 12 cohorts. (PDF) [file pone.0182472.s002.pdf]

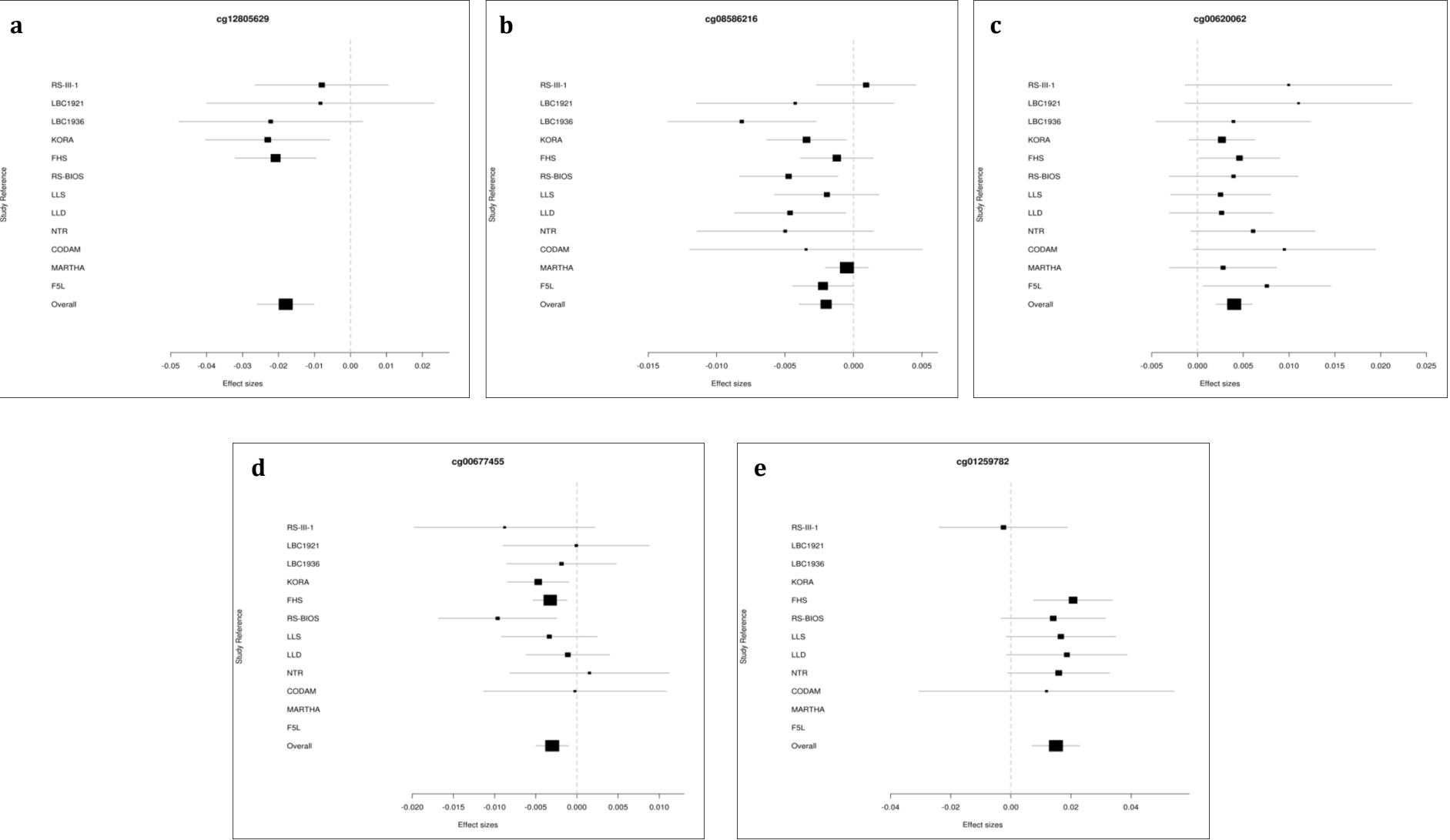

**S3 (a-e) Figs. Forest plots.** *Trans*-meQTLs of the Genetic risk score model across 12 cohorts.

Supplement: S3 Fig — Trans-meQTLs of the Genetic risk score model across 12 cohorts. (PDF) [file pone.0182472.s003.pdf]
