# Supplementary material for: Genetically defined elevated homocysteine levels do not result in widespread changes of DNA methylation in leukocytes
Source: PLoS One. 2017 Oct 30;12(10):e0182472. doi: 10.1371/journal.pone.0182472 (PMC5662081; doi:10.1371/journal.pone.0182472)
Supplement: S4 Fig — (a) MTHFR 677 C>T variant or (b) Genetic risk score associated 3 DMRs of IGF2/H19 genes containing 7 CpGs (green) from the 450k data; DMR0 “Chr.11:2,170,380–2,170,517” with 2 CpGs, DMR2 “Chr.11:2,154,113–2,154,414” with 4 CpGs and H19-DMR3 “Chr.11:2,021,072–2,021,273” with 1 CpG. (PDF) [file pone.0182472.s004.pdf]

a

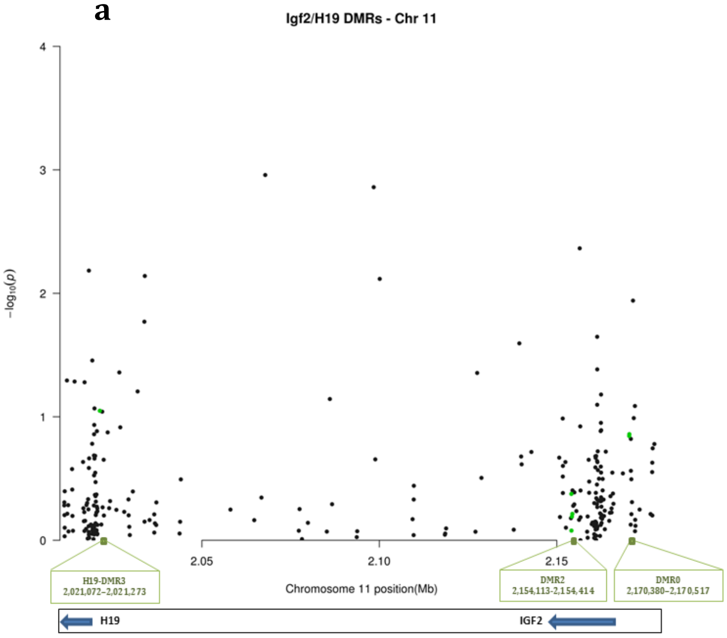

b

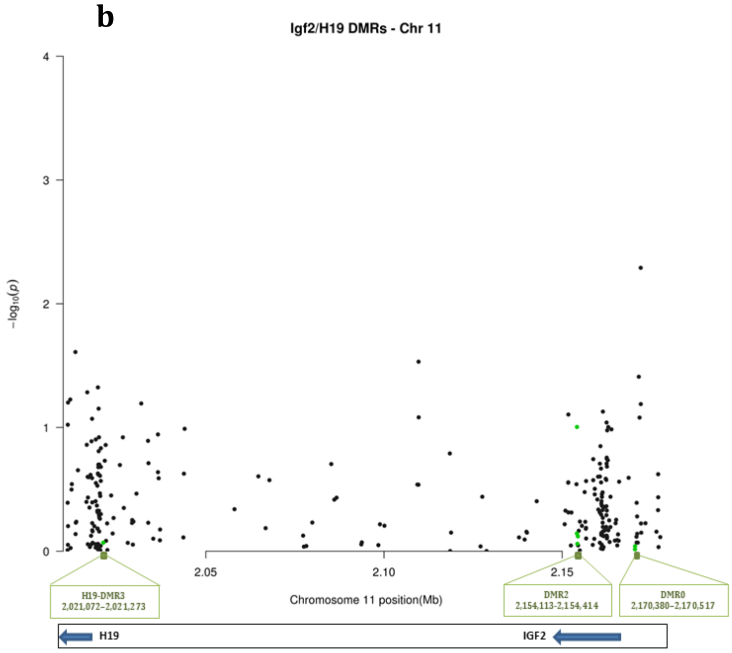

S4 (a and b) Figs. Regional manhattan plots. (a) MTHFR 677 C>T variant or (b) Genetic risk score associated 3 DMRs of IGF2/H19 genes containing 7 CpGs (green) from the 450k data; DMR0 "Chr.11:2,170,380-2,170,517" with 2 CpGs, DMR2 "Chr.11:2,154,113-2,154,414" with 4 CpGs and H19-DMR3 "Chr.11:2,021,072-2,021,273" with 1 CpG.
