# Supplementary material for: Genetically defined elevated homocysteine levels do not result in widespread changes of DNA methylation in leukocytes
Source: PLoS One. 2017 Oct 30;12(10):e0182472. doi: 10.1371/journal.pone.0182472 (PMC5662081; doi:10.1371/journal.pone.0182472)
Supplement: S1 Table — (PDF) [file pone.0182472.s005.pdf]

S1 Table. Cohort characteristics

Sr. No.	Study Cohorts	N	Women [%]	MTHFR Genotype		GRS2 [Mean (S.D.)]	Age [Mean (S.D.)]	Gran [Mean (S.D.)]			Lym [Mean (S.D.)]				Mono [Mean (S.D.)]	Study Design	Pathophysiology
				%CT	%TT												
	**CHARGE consortium**																
1	RS-III-I	729	54.0	41.6 (303)	8.6 (63)	1.09 (0.19)	59.86 (8.16)	4.21 (1.51)			2.42 (0.67)				0.42 (0.42)	Prospective, population-based	Mainly healthy
2	LBC1921	436	60.3	45.0 (196)	12.4 (24)	1.13 (0.19)	79.10 (0.60)	4.6 (1.34)	0.17 (0.17)	0.02 (0.04)	1.71 (1.12)				0.54 (0.18)	Prospective, population-based	Mainly healthy
3	LBC1936	905	49.4	43.2 (391)	11.4 (103)	1.17 (0.18)	69.60 (0.80)	4.5 (1.54)	0.17 (0.13)	0.04 (0.04)	1.90 (1.43)				0.53 (0.19)	Prospective, population-based	Mainly healthy
4	KORA	1,799	51.1	45 (779)	10.6 (183)	1.11 (0.18)	60.98 (8.89)	0.33 (0.09)			0.10[W] (0.07)	0.16[X] (0.06)	0.02[Y] (0.02)	0.05[Z] (0.03)	0.12 (0.03)	Prospective, population-based	Mainly healthy
5	FHS	2,408	54.3	58.9 (1206)	18.2 (306)	1.12 (0.17)	66.4 (8.93)	0.49 (0.09)			0.10[W] (0.06)	0.19[X] (0.06)	0.02[Y] (0.02)	0.04[Z] (0.03)	0.13 (0.03)	Prospective, population-based	Mainly healthy
	**BIOS consortium**																
6	RS-II-3/ RS-III-2	722	57.8	43.2 (312)	12.3 (89)	1.15 (0.19)	67.60 (5.98)	48.32[N] (7.04)	2.74[E] (0.73)	0.68[B] (0.18)	39.66 (9.71)				7.19 (1.30)	Prospective, population-based	Mainly healthy
7	LLS	739	51.7	48.0 (355)	11.8 (87)	1.14 (0.18)	58.98 (6.70)	58.14[N] (7.93)	2.63[E] (1.17)	0.64[B] (0.25)	29.52 (6.18)				5.68 (1.16)	Prospective, family-based	Mainly healthy
8	LLD	744	57.8	44.2 (329)	9.9 (74)	1.13 (0.17)	45.56 (13.32)	54.15[N] (7.22)	2.93[E] (1.49)	0.54[B] (0.25)	33.47 (7.10)				8.14 (1.53)	Prospective, population-based	-
9	NTR	691	68.5	41.8 (289)	10.3 (71)	1.12 (0.18)	33.95 (12.11)	52.59[N] (7.66)	2.81[E] (1.21)	0.47[B] (0.41)	35.11 (7.81)				7.72 (2.08)	-	-
10	CODAM	161	45.3	43.5 (70)	12.4 (20)	1.14 (0.17)	65.40 (6.77)	40.67[N] (6.97)	2.80[E] (0.63)	0.75[B] (0.20)	51.11 (14.06)				7.95 (1.63)	Prospective, Observational	Mildly increased DM2 /CVD risk factors
	**Other cohorts**																
11	MARTHA	349	78.5	48.1 (168)	11.7 (41)	1.14 (0.18)	43.77 (14.09)	4.58 (0.38)			1.87 (0.62)				0.32 (0.13)	Retrospective	Patients with Venous Thromboembolism
12	F5L	211	52.1	48.8 (103)	20.9 (44)	1.15 (0.18)	39.7 (16.80)	0.56 (0.13)			0.04 [W] (0.04)	0.22 [X] (0.08)	0.10 [Y] (0.05)	0.10 [Z] (0.03)	0.08 (0.03)	Pedigree-based	Mainly healthy, probands with VTE

[N] Neutrophils, [E] Eosinophils, [B] Basophils (Imputed)
[W] CD8T, [X] CD4T, [Y] NK, [Z] Bcell (Imputed)
