# Supplementary material for: Genetically defined elevated homocysteine levels do not result in widespread changes of DNA methylation in leukocytes
Source: PLoS One. 2017 Oct 30;12(10):e0182472. doi: 10.1371/journal.pone.0182472 (PMC5662081; doi:10.1371/journal.pone.0182472)
Supplement: S2 Table — Quality control of SNPs and imputation. (PDF) [file pone.0182472.s006.pdf]

S2 Table. Details of genotyping methods: Quality control of SNPs and imputation

		Genotyping					Imputation		
		Platform	Inclusion criteria			Imputation panel	Imputation software	Inclusion criteria	
**Sr. No.**	**Cohort**		**MAF**	**Call rate**	**p for HWE**			**MAF**	**Imputation quality**
	**CHARGE consortium**								
1	RS-III-I	Illumina / HumanHap 610 QUAD	≥1%	≥97.5%	$>10^{-6}$	1000G	MACH	≥1%	R2≥0.3
2	LBC1921	Illumina / HumanHap 610 QUAD	≥1%	≥95%	$>10^{-3}$	1000G	MACH	>0%	Uploaded without filtering
3	LBC1936	Illumina / HumanHap 610 QUAD	≥1%	≥95%	$>10^{-3}$	1000G	MACH	>0%	Uploaded without filtering
4	KORA	Affymetrix Axiom	≥1%	≥98%	$>5*10^{-6}$	1000G	IMPUTE v2.3.0	≥1%	NA
5	FHS	Affymetrix 500K Dual	≥1%	≥98%	$>5*10^{-6}$	1000G	MACH	≥1%	$R^2 \geq 0.3$
	**BIOS consortium**								
6	RS-II-3/ RS-III-2	Illumina / HumanHap 550 V.3 DUO, Illumina / HumanHap 610 QUAD	≥5%	≥95%	$>10^{-4}$	GoNL	IMPUTE2	≥5%	Info-score ≥0.5
7	LLS	Illumina Infinium HD Human660W-Quad, Illumina Infinium HD Human OmniExpress	≥5%	≥95%	$>10^{-4}$	GoNL	IMPUTE2	≥5%	Info-score ≥0.5
8	LLD	Illumina Cyto SNP12 v2 & Illumina ImmunoChip	≥5%	≥95%	$>10^{-4}$	GoNL	IMPUTE2	≥5%	Info-score ≥0.5
9	NTR	Affy6, Epigen	≥5%	≥95%	$>10^{-4}$	GoNL	IMPUTE2	≥5%	Info-score ≥0.5
10	CODAM	Illumina Human Omni Express	≥5%	≥95%	$>10^{-4}$	GoNL	IMPUTE2	≥5%	Info-score ≥0.5
	**Other cohorts**								
11	MARTHA	Illumina Human 660W-Quad BeadChip, Illumina Human 610-Quad BeadChip	≥1%	≥95%	none	1000G	MACH, Minimac	>1%	$R^2 > 0.3$
12	F5L	Illumina Human 660W-Quad BeadChip	≥ 20 (count)	≥95%	None	1000G	IMPUTE2	>0%	No filtering
