# Supplementary material for: Genetically defined elevated homocysteine levels do not result in widespread changes of DNA methylation in leukocytes
Source: PLoS One. 2017 Oct 30;12(10):e0182472. doi: 10.1371/journal.pone.0182472 (PMC5662081; doi:10.1371/journal.pone.0182472)
Supplement: S3 Table — The CpGs are sorted in base pairs. (PDF) [file pone.0182472.s007.pdf]

S3 Table. 35 *cis*-meQTLs with FDR<0.05 that are associated with MTHFR 677C>T variant (rs1801133) in a samples size of 9,894. The CpGs are sorted in base pairs.

Rank	CpG	Sample Size	Effect	StdErr	Pvalue	FDR	HetISq	HetPVal	Genes Associated	Chr	Bp	Enhancer	Promoter
22	cg16429182	6,837	0.003	0.000	1.23E-15	2.60E-11	68.7	3.92E-03	-	1	11824095	-	-
32	cg15568408	9,334	-0.002	0.000	2.49E-08	3.62E-04	44.9	6.02E-02	AGTRAP (+29763), MTHFR (+40255)	1	11825928	-	-
5	cg06770735	9,894	0.009	0.000	9.86E-104	9.18E-99	83.7	3.58E-10	MTHFR (+26104), AGTRAP (+43914)	1	11840031	-	-
18	cg02448276	9,894	0.002	0.000	4.98E-23	1.29E-18	60.9	3.09E-03	MTHFR (+20897), AGTRAP (+49121)	1	11845238	-	-
11	cg21864959	9,894	-0.006	0.000	1.22E-50	5.16E-46	78.0	6.55E-07	MTHFR (+18272), AGTRAP (+51746)	1	11847863	TRUE	-
30	cg18276943	9,894	0.001	0.000	1.60E-09	2.48E-05	41.2	6.63E-02	MTHFR (+12573), AGTRAP (+57445)	1	11853610	TRUE	-
1	cg17514528	9,894	0.020	0.001	1.00E-154	4.66E-149	79.2	1.96E-07	CLCN6 (-3275), MTHFR (+3228)	1	11862907	-	-
4	cg17745097	9,894	0.007	0.000	1.34E-113	1.56E-108	46.8	3.69E-02	CLCN6 (-2817), MTHFR (+2770)	1	11863365	-	TRUE
12	cg05228408	9,894	-0.007	0.001	9.22E-43	3.58E-38	22.5	2.22E-01	CLCN6 (-878), MTHFR (+831)	1	11865352	-	-
31	cg01179095	9,894	0.002	0.000	1.35E-08	2.03E-04	0.0	7.53E-01	NPPA (+7211), CLCN6 (+34422)	1	11900652	-	-
10	cg20202438	9,894	0.008	0.001	1.87E-60	8.71E-56	61.6	2.57E-03	NPPA (-123)	1	11907986	TRUE	-
8	cg25341925	9,894	0.007	0.000	2.36E-67	1.37E-62	55.1	1.08E-02	NPPA (-243)	1	11908058	TRUE	-
6	cg05396397	9,894	0.017	0.001	5.99E-95	4.65E-90	43.6	5.27E-02	NPPA (-301)	1	11908164	TRUE	-
3	cg06193043	9,894	0.024	0.001	2.89E-117	4.49E-112	68.5	2.58E-04	NPPA (-384)	1	11908199	TRUE	-
7	cg14194983	9,894	0.014	0.001	5.30E-85	3.53E-80	62.0	2.29E-03	NPPA (-483)	1	11908298	TRUE	-
2	cg24844545	9,894	0.021	0.001	1.10E-131	2.56E-126	64.2	1.24E-03	NPPA (-484)	1	11908347	TRUE	-
17	cg07585427	8,553	0.010	0.001	1.72E-24	4.71E-20	36.4	1.17E-01	NPPA (-9687), NPPB (+1465)	1	11917550	-	-
25	cg24697075	9,894	0.005	0.001	1.30E-12	2.42E-08	0.0	6.31E-01	NPPB (+160)	1	11918807	-	-
16	cg00299820	9,334	-0.006	0.000	1.36E-38	3.96E-34	0.0	4.88E-01	NPPB (-24139), KIAA2013 (+43354)	1	11943154	TRUE	-
15	cg03321020	9,894	-0.010	0.001	4.53E-40	1.41E-35	0.0	8.43E-01	NPPB (-39359), KIAA2013 (+28134)	1	11958326	-	-
9	cg27432085	9,894	-0.017	0.001	4.25E-62	2.20E-57	16.8	2.79E-01	NPPB (-39557), KIAA2013 (+27936)	1	11958572	-	-
23	cg09872491	9,894	0.003	0.000	4.59E-14	9.29E-10	11.0	3.37E-01	NPPB (-40174), KIAA2013 (+27319)	1	11959141	-	-
19	cg23086274	9,173	-0.003	0.000	1.99E-20	4.88E-16	79.3	5.66E-06	NPPB (-49192), KIAA2013 (+18301)	1	11968159	-	TRUE
34	cg03532363	9,334	-0.002	0.000	1.27E-06	1.69E-02	0.0	6.24E-01	NPPB (-50656), KIAA2013 (+16837)	1	11969623	-	TRUE
26	cg22932223	6,277	0.006	0.001	1.40E-12	2.51E-08	0.0	4.18E-01	-	1	11986734	-	TRUE
13	cg07603449	6,837	0.012	0.001	2.26E-42	8.10E-38	29.4	2.04E-01	-	1	11986842	-	TRUE

27	cg10438121	9,334	0.007	0.001	2.20E-11	3.79E-07	0.0	6.29E-01	PLOD1 (-3032)	1	11991667	-	-
21	cg03529003	9,894	0.004	0.000	2.45E-18	5.43E-14	49.2	2.72E-02	PLOD1 (-466)	1	11994281	-	TRUE
33	cg18184769	9,894	0.003	0.001	2.65E-07	3.74E-03	5.8	3.89E-01	MFN2 (-499)	1	12039714	-	TRUE
28	cg03593358	9,894	0.003	0.000	1.48E-10	2.46E-06	0.0	8.56E-01	MFN2 (-276)	1	12039985	-	TRUE
24	cg05398737	8,643	0.004	0.001	3.49E-13	6.77E-09	0.0	8.44E-01	TNFRSF8 (-44654), MFN2 (+38542)	1	12078755	-	-
29	cg22534105	9,894	0.003	0.001	2.11E-10	3.39E-06	31.4	1.40E-01	TNFRSF8 (-44621), MFN2 (+38575)	1	12078836	-	-
14	cg02156723	9,894	-0.008	0.001	1.99E-41	6.62E-37	53.3	1.47E-02	TNFRSF8 (-22778), MFN2 (+60418)	1	12100631	TRUE	-
20	cg22877366	9,894	-0.008	0.001	1.36E-19	3.17E-15	43.0	5.59E-02	TNFRSF8 (-15789), MFN2 (+67407)	1	12107668	-	TRUE
35	cg20978923	6,837	0.004	0.001	2.85E-06	3.69E-02	40.7	1.20E-01	-	1	12184574	-	-

Effect: Regression coefficients

FDR: False discovery Rate adjusted P-value, threshold = 0.05

HetISq: Heterogeneity I^2 parameter

HetPVal: Heterogeneity p-value

Enhancer & promoter annotations from Illumina 450k annotation
