# Supplementary material for: Genetically defined elevated homocysteine levels do not result in widespread changes of DNA methylation in leukocytes
Source: PLoS One. 2017 Oct 30;12(10):e0182472. doi: 10.1371/journal.pone.0182472 (PMC5662081; doi:10.1371/journal.pone.0182472)
Supplement: S4 Table — The CpGs are sorted in chromosomes and base pairs. (PDF) [file pone.0182472.s008.pdf]

S4 Table. 113 *cis*-meQTLs with FDR<0.05 that are associated with Genetic Risk Score of 18 Hcy-associated variants in a sample size of 9,894.

The CpGs are sorted in chromosomes and base pairs.

Rank	CpG	Sample Size	Effect	StdErr	Pvalue	FDR	HetISq	HetPVal	Genes Associated	Chr	Bp	Enhancer	Promoter
43	cg17514528	9,894	0.022	0.003	6.43E-14	6.51E-10	25	1.99E-01	CLCN6 (-3275), MTHFR (+3228)	1	11862907	-	-
78	cg17745097	9,894	0.006	0.001	1.93E-07	1.06E-03	7.7	3.70E-01	CLCN6 (-2817), MTHFR (+2770)	1	11863365	-	TRUE
31	cg05228408	9,894	-0.016	0.002	4.45E-18	6.28E-14	0	5.19E-01	CLCN6 (-878), MTHFR (+831)	1	11865352	-	-
25	cg20202438	9,894	0.016	0.002	2.77E-21	4.96E-17	57	7.45E-03	NPPA (-123)	1	11907986	TRUE	-
24	cg25341925	9,894	0.014	0.001	2.23E-21	4.15E-17	20.2	2.45E-01	NPPA (-243)	1	11908058	TRUE	-
18	cg05396397	9,894	0.031	0.003	1.74E-26	4.26E-22	8.3	3.64E-01	NPPA (-301)	1	11908164	TRUE	-
7	cg06193043	9,894	0.049	0.004	4.14E-39	2.41E-34	69.8	1.43E-04	NPPA (-384)	1	11908199	TRUE	-
16	cg14194983	9,894	0.029	0.003	2.05E-27	5.62E-23	64.4	1.16E-03	NPPA (-483)	1	11908298	TRUE	-
4	cg24844545	9,894	0.050	0.003	5.25E-54	6.11E-49	67.9	3.30E-04	NPPA (-484)	1	11908347	TRUE	-
26	cg00299820	9,334	-0.014	0.002	1.91E-20	3.29E-16	55.5	1.66E-02	NPPB (-24139), KIAA2013 (+43354)	1	11943154	TRUE	-
63	cg03321020	9,894	-0.016	0.003	5.19E-09	3.50E-05	20	2.47E-01	NPPB (-39359), KIAA2013 (+28134)	1	11958326	-	-
23	cg27432085	9,894	-0.037	0.004	3.21E-22	6.23E-18	34.9	1.11E-01	NPPB (-39557), KIAA2013 (+27936)	1	11958572	-	-
79	cg23086274	9,173	-0.006	0.001	2.02E-07	1.09E-03	70.1	7.72E-04	NPPB (-49192), KIAA2013 (+18301)	1	11968159	-	TRUE
69	cg03532363	9,334	-0.009	0.002	2.14E-08	1.33E-04	35.8	1.22E-01	NPPB (-50656), KIAA2013 (+16837)	1	11969623	-	TRUE
32	cg07603449	6,837	0.027	0.003	1.31E-17	1.79E-13	0	4.47E-01	-	1	11986842	-	TRUE
54	cg02156723	9,894	-0.014	0.002	1.77E-10	1.37E-06	56.8	7.87E-03	TNFRSF8 (-22778), MFN2 (+60418)	1	12100631	TRUE	-
29	cg11803389	6,837	0.051	0.006	3.42E-18	5.14E-14	4.5	3.92E-01	-	1	45965020	-	TRUE
46	cg17297354	9,894	0.007	0.001	8.83E-13	8.22E-09	33.4	1.23E-01	RYR2 (-149036), MTR (+98085)	1	237056641	TRUE	-
112	cg07061783	6,277	-0.018	0.004	1.40E-05	4.72E-02	0	9.66E-01	HOXB9 (-1827)	6	25882402	-	-
101	cg03264133	9,894	-0.040	0.009	2.62E-06	1.04E-02	15.4	2.93E-01	SLC17A3 (-8017), SLC17A2 (+48458)	6	25882463	-	-
93	cg12310025	9,894	-0.050	0.010	1.67E-06	7.27E-03	33.5	1.22E-01	SLC17A3 (-8035), SLC17A2 (+48440)	6	25882481	-	-
92	cg03517284	9,894	-0.041	0.009	1.31E-06	5.76E-03	21.4	2.33E-01	SLC17A3 (-8096), SLC17A2 (+48379)	6	25882590	-	-
108	cg08501292	6,754	0.006	0.001	8.40E-06	3.01E-02	18.6	2.77E-01	TRIM38 (-107)	6	25962987	TRUE	TRUE
104	cg06035815	9,894	0.008	0.002	4.73E-06	1.81E-02	0	7.06E-01	HIST1H4D (-270)	6	26189597	-	TRUE
39	cg14345882	9,894	0.039	0.005	8.29E-15	9.19E-11	0	6.18E-01	BTN3A2 (-3636)	6	26364793	TRUE	TRUE
102	cg27626102	9,334	-0.008	0.002	3.62E-06	1.40E-02	0	6.54E-01	STEAP2 (-244)	7	89840731	-	-

96	cg27367526	9,894	-0.014	0.003	1.69E-06	7.09E-03	10.6	3.42E-01	STEAP2 (+717)	7	89841692	-	-
94	cg01887633	6,754	0.009	0.002	1.67E-06	7.20E-03	0	5.56E-01	GTPBP10 (-26371), C7orf63 (+75120)	7	89949631	-	-
91	cg25739043	9,894	-0.010	0.002	1.26E-06	5.59E-03	0	5.35E-01	GTPBP10 (-25496), C7orf63 (+75995)	7	89950458	-	-
88	cg12445565	9,894	0.014	0.003	1.04E-06	4.75E-03	38.5	8.44E-02	GTPBP10 (-24264), C7orf63 (+77227)	7	89951738	-	-
75	cg25638870	7,535	0.027	0.005	1.44E-07	8.18E-04	0	6.56E-01	NOX4 (-89)	11	89224717	-	-
107	cg25542007	6,277	-0.060	0.013	6.51E-06	2.41E-02	0	5.66E-01	FLRT1 (-18527), OTUB1 (+99510)	11	89323282	-	-
109	cg05041596	8,095	-0.026	0.006	9.32E-06	3.26E-02	9.7	3.51E-01	NAALAD2 (-408)	11	89867385	-	-
113	cg21721566	9,894	0.014	0.003	1.53E-05	4.98E-02	32.9	1.27E-01	ACADS (-402)	12	121163144	TRUE	TRUE
81	cg13567169	9,894	-0.014	0.003	2.35E-07	1.22E-03	35.5	1.06E-01	SPPL3 (-68677), HNF1A (-5717)	12	121410855	-	-
59	cg14101638	8,095	0.009	0.002	3.44E-09	2.46E-05	22.2	2.32E-01	HNF1A (+88)	12	121416612	-	-
111	cg25615068	9,894	-0.011	0.003	1.15E-05	3.97E-02	54.6	1.19E-02	HNF1A (+26737), OASL (+33494)	12	121443309	-	-
106	cg02403541	9,894	0.002	0.000	6.29E-06	2.34E-02	55.9	9.26E-03	OASL (+22467), HNF1A (+37764)	12	121454288	-	TRUE
51	cg19788934	6,837	-0.018	0.003	9.92E-11	8.10E-07	0	8.48E-01	MSRA (-149089), TNKS (+349296)	12	121455706	-	-
62	cg08335008	9,334	0.027	0.005	4.52E-09	3.10E-05	41	8.45E-02	CDH15 (-12643), ACSF3 (+65303)	16	89225495	-	-
97	cg02648441	9,894	-0.008	0.002	2.11E-06	8.77E-03	0	8.30E-01	CDH15 (+4689), SLC22A31 (+23677)	16	89242827	-	-
66	cg09164168	9,894	0.015	0.003	1.32E-08	8.54E-05	44.1	4.98E-02	ZNF778 (+100635), ANKRD11 (+172223)	16	89384721	-	-
73	cg26536240	9,894	0.014	0.003	8.65E-08	5.04E-04	2.1	4.23E-01	ANKRD11 (+47232), ZNF778 (+225626)	16	89509760	TRUE	-
76	cg15206445	9,894	-0.012	0.002	1.63E-07	9.15E-04	19.9	2.48E-01	SPG7 (-1468)	16	89573312	TRUE	-
105	cg16428612	9,894	-0.004	0.001	6.29E-06	2.36E-02	22.2	2.25E-01	SPG7 (-1293)	16	89573535	-	-
77	cg07924503	9,894	-0.016	0.003	1.85E-07	1.03E-03	20.3	2.44E-01	SPG7 (-1197)	16	89573631	-	-
50	cg02244288	9,894	-0.012	0.002	5.00E-11	4.23E-07	8.7	3.60E-01	SPG7 (-873)	16	89573955	-	-
95	cg08845973	9,894	-0.010	0.002	1.69E-06	7.15E-03	16.8	2.79E-01	RPL13 (-35017), SPG7 (+17243)	16	89592071	-	-
99	cg03169557	9,894	-0.011	0.002	2.45E-06	9.92E-03	0	5.04E-01	RPL13 (-28090), SPG7 (+24170)	16	89598950	-	-
82	cg08784311	5,330	0.053	0.011	4.22E-07	2.16E-03	0	5.05E-01	CPNE7 (-9427), RPL13 (+5684)	16	89632724	-	-
60	cg16616467	9,894	-0.013	0.002	3.77E-09	2.66E-05	15.8	2.89E-01	CPNE7 (+715)	16	89642914	-	-
55	cg26217441	9,894	-0.016	0.003	2.65E-10	2.02E-06	23.4	2.13E-01	DPEP1 (-43561), CPNE7 (+1263)	16	89643414	-	-
84	cg04080022	9,894	0.010	0.002	4.79E-07	2.37E-03	0	8.36E-01	DPEP1 (-38489), CPNE7 (+6335)	16	89648534	TRUE	-
110	cg02628002	9,894	-0.003	0.001	1.04E-05	3.61E-02	9.9	3.48E-01	DPEP1 (-37367), CPNE7 (+7457)	16	89649608	-	-
58	cg02848750	9,894	-0.010	0.002	2.11E-09	1.54E-05	36.3	1.01E-01	DPEP1 (-18569), CPNE7 (+26255)	16	89668406	TRUE	-
1	cg01097406	9,894	0.232	0.011	9.57E-104	4.46E-98	81.3	1.58E-08	-	16	89675127	-	-

103	cg20972466	9,334	0.010	0.002	4.41E-06	1.70E-02	10.4	3.47E-01	DPEP1 (-7380), CPNE7 (+37444)	16	89679595	-	-
42	cg06082883	9,894	-0.015	0.002	6.40E-14	6.62E-10	54.2	1.26E-02	DPEP1 (-279)	16	89686744	-	-
71	cg05059843	9,894	0.007	0.001	5.76E-08	3.48E-04	16.8	2.79E-01	DPEP1 (-62)	16	89686913	-	-
13	cg01749904	9,334	0.042	0.004	1.08E-30	3.59E-26	65.3	2.07E-03	DPEP1 (+29)	16	89687052	-	-
83	cg00996377	9,894	0.004	0.001	4.23E-07	2.14E-03	58	6.03E-03	DPEP1 (+2782), CHMP1A (+34347)	16	89689757	-	-
15	cg02393727	9,334	-0.043	0.004	9.36E-29	2.72E-24	66.5	1.45E-03	DPEP1 (+3113), CHMP1A (+34016)	16	89690088	-	-
9	cg05347456	9,334	-0.047	0.004	1.01E-34	4.70E-30	75.6	2.70E-05	DPEP1 (+3287), CHMP1A (+33842)	16	89690262	-	-
48	cg09869502	9,894	-0.015	0.002	1.18E-11	1.04E-07	69.3	1.84E-04	DPEP1 (+3827), CHMP1A (+33302)	16	89690802	TRUE	-
49	cg01747591	9,894	0.008	0.001	2.09E-11	1.80E-07	37.4	9.18E-02	DPEP1 (+16589), CHMP1A (+20540)	16	89703612	-	-
44	cg06047934	9,894	0.007	0.001	1.22E-13	1.18E-09	29.3	1.58E-01	CHMP1A (+11635), DPEP1 (+25494)	16	89712517	-	-
17	cg27134767	9,894	0.020	0.002	8.62E-27	2.23E-22	51.6	1.92E-02	CHMP1A (+10765), DPEP1 (+26364)	16	89713339	-	-
98	cg26681017	9,894	0.015	0.003	2.41E-06	9.84E-03	7.7	3.70E-01	-	16	89723193	-	-
8	cg07835443	6,837	0.050	0.004	1.17E-38	6.05E-34	38.8	1.33E-01	ABL1 (-48359), EXOSC2 (+93314)	16	89734986	-	-
10	cg16725984	9,334	0.048	0.004	3.03E-34	1.28E-29	62.7	4.10E-03	CDK10 (-17867), CHMP1A (-11080)	16	89735184	-	-
28	cg00574278	9,894	0.008	0.001	1.36E-18	2.11E-14	11.5	3.33E-01	CDK10 (-17221), CHMP1A (-11726)	16	89735878	-	-
100	cg16570414	9,894	0.005	0.001	2.61E-06	1.05E-02	16.2	2.85E-01	CDK10 (-16261), CHMP1A (-12686)	16	89736790	TRUE	-
5	cg03605463	9,894	0.074	0.005	1.86E-49	1.44E-44	51.1	2.10E-02	CHMP1A (-16460), CDK10 (-12487)	16	89740564	-	-
14	cg09827496	6,837	0.049	0.004	2.16E-30	6.71E-26	0	5.93E-01	GALNT9 (+53539), NOC4L (+223373)	16	89740639	-	-
35	cg04747957	6,837	-0.004	0.000	2.32E-16	2.84E-12	74.2	7.07E-04	ENPP5 (-149975), RCAN2 (+170368)	16	89751896	-	-
2	cg04155793	9,894	0.054	0.003	3.46E-61	8.06E-56	82.3	3.46E-09	CDK10 (-204)	16	89752847	-	TRUE
3	cg12119029	9,894	0.047	0.003	1.59E-55	2.47E-50	72.9	2.87E-05	CDK10 (-220)	16	89752879	-	TRUE
56	cg00796364	9,334	0.004	0.001	6.63E-10	4.98E-06	28.7	1.80E-01	CDK10 (+473)	16	89753572	-	TRUE
53	cg27113347	9,894	-0.014	0.002	1.76E-10	1.39E-06	57.5	6.73E-03	CDK10 (+7534), C16orf7 (+26784)	16	89760585	-	-
36	cg04544017	9,894	-0.011	0.001	3.03E-16	3.62E-12	8.6	3.61E-01	CDK10 (+10271), C16orf7 (+24047)	16	89763370	-	-
74	cg26822161	9,894	-0.002	0.000	1.23E-07	7.07E-04	62.9	1.80E-03	C16orf7 (+14805), CDK10 (+19513)	16	89772612	-	-
6	cg00261912	8,095	-0.046	0.004	6.64E-40	4.42E-35	69	3.58E-04	C16orf7 (+9654), CDK10 (+24664)	16	89777763	-	-
41	cg03809021	9,894	-0.030	0.004	3.33E-14	3.52E-10	27.6	1.73E-01	ZNF276 (+43196), FANCA (+51917)	16	89831123	-	-
40	cg05795554	8,095	-0.009	0.001	2.69E-14	2.91E-10	48.1	3.70E-02	FANCA (-124)	16	89883212	-	TRUE
57	cg04287289	8,095	-0.014	0.002	1.11E-09	8.21E-06	42.3	6.73E-02	FANCA (-152)	16	89883240	-	TRUE
65	cg26513180	8,095	-0.011	0.002	7.55E-09	4.95E-05	49.7	3.04E-02	SCNN1A (-2185)	16	89883248	-	TRUE

70	cg05139061	9,894	-0.010	0.002	2.18E-08	1.34E-04	5.3	3.93E-01	SPIRE2 (-3395)	16	89891535	-	-
38	cg07402062	9,894	-0.035	0.005	5.87E-15	6.67E-11	28.7	1.64E-01	SPIRE2 (-832)	16	89894098	-	-
19	cg21285383	9,894	-0.022	0.002	2.58E-26	6.01E-22	50.6	2.24E-02	SPIRE2 (-622)	16	89894308	-	-
11	cg03388025	9,894	-0.035	0.003	3.75E-32	1.46E-27	59.8	4.05E-03	SPIRE2 (-601)	16	89894329	-	-
68	cg05500240	9,894	-0.011	0.002	1.97E-08	1.24E-04	56.1	8.97E-03	TCF25 (-14360), SPIRE2 (+30727)	16	89925609	-	-
85	cg05941025	9,894	-0.011	0.002	5.21E-07	2.53E-03	54.7	1.17E-02	TCF25 (-12373), SPIRE2 (+32714)	16	89927596	-	-
67	cg19635926	6,277	-0.009	0.002	1.50E-08	9.57E-05	11	3.43E-01	-	16	89946313	-	-
37	cg06558623	6,837	-0.022	0.003	5.93E-16	6.90E-12	20.4	2.74E-01	BSPH1 (-2629), ELSPBP1 (+148)	16	89946397	-	-
61	cg09569215	9,894	0.010	0.002	4.36E-09	3.03E-05	38.6	8.36E-02	TUBB3 (-22969), TCF25 (+26724)	16	89966693	-	-
86	cg06017697	9,894	0.007	0.001	7.00E-07	3.29E-03	0	6.63E-01	TUBB3 (-12995), TCF25 (+36698)	16	89976667	TRUE	-
21	cg05749642	9,334	-0.019	0.002	6.35E-24	1.34E-19	30.4	1.66E-01	TUBB3 (-7442), TCF25 (+42251)	16	89982268	-	-
34	cg01768446	9,894	-0.012	0.002	1.09E-16	1.37E-12	46.1	4.00E-02	TUBB3 (-7243), TCF25 (+42450)	16	89982419	-	-
27	cg01023759	5,038	0.050	0.006	1.12E-18	1.80E-14	73.1	2.30E-03	-	16	89983766	-	-
33	cg08627397	5,472	0.043	0.005	7.50E-17	9.98E-13	54.8	5.03E-02	TUBB3 (-5634), TCF25 (+44059)	16	89984028	-	-
80	cg00534858	6,277	0.022	0.004	2.33E-07	1.22E-03	44.3	1.27E-01	-	16	90074520	-	-
64	cg06907930	7,535	-0.052	0.009	6.49E-09	4.32E-05	0	4.46E-01	C16orf3 (-14514), LOC100130015 (+3368)	16	90110798	-	-
87	cg07800524	9,894	0.007	0.002	9.19E-07	4.24E-03	0	5.36E-01	FUT1 (-830), FGF21 (+329)	19	49259452	TRUE	-
12	cg15157241	9,334	-0.052	0.004	4.39E-32	1.57E-27	73.4	9.53E-05	PKNOX1 (+1586), CBS (+100243)	21	44396204	-	-
22	cg08751406	9,334	0.027	0.003	9.79E-23	1.98E-18	17.8	2.79E-01	CBS (+25595), PKNOX1 (+76234)	21	44470900	-	-
89	cg07211972	8,095	0.014	0.003	1.05E-06	4.75E-03	0	7.50E-01	CBS (+22999), PKNOX1 (+78830)	21	44473496	-	-
52	cg01788910	9,894	0.022	0.003	1.16E-10	9.31E-07	0	4.63E-01	CBS (+22927), PKNOX1 (+78902)	21	44473568	-	-
72	cg14171527	9,894	-0.010	0.002	6.32E-08	3.77E-04	43.3	5.41E-02	CBS (+22655), PKNOX1 (+79174)	21	44473840	-	-
30	cg19312314	9,334	0.034	0.004	3.56E-18	5.18E-14	0	6.34E-01	CBS (+22485), PKNOX1 (+79344)	21	44473962	-	-
47	cg02644491	9,894	0.021	0.003	3.09E-12	2.82E-08	0	6.26E-01	CBS (+22428), PKNOX1 (+79401)	21	44474019	-	-
90	cg09128751	9,894	0.006	0.001	1.13E-06	5.06E-03	21.4	2.33E-01	CBS (+10290), PKNOX1 (+91539)	21	44486205	-	-
45	cg22633722	9,894	-0.012	0.002	8.49E-13	8.07E-09	16.1	2.86E-01	CBS (-296)	21	44496743	-	-
20	cg15300856	9,894	-0.015	0.001	3.18E-26	7.05E-22	68.7	2.35E-04	-	21	44497019	-	-

Effect: Regression coefficients

FDR: False discovery Rate adjusted P-value, threshold = 0.05

HetISq: Heterogeneity I^2 parameter

HetPVal: Heterogeneity p-value

Enhancer & promoter annotations from Illumina 450k annotation
