# Supplementary material for: Genetically defined elevated homocysteine levels do not result in widespread changes of DNA methylation in leukocytes
Source: PLoS One. 2017 Oct 30;12(10):e0182472. doi: 10.1371/journal.pone.0182472 (PMC5662081; doi:10.1371/journal.pone.0182472)
Supplement: S5 Table — (PDF) [file pone.0182472.s009.pdf]

S5 Table. *Trans*-meQTLs with FDR<0.05 that are associated with MTHFR 677C>T variant (rs1801133) in a sample size of 9,894.

Rank	CpG	N	Effect	StdErr	Pvalue	FDR	HetISq	HetPVal	Genes Associated	Chr	Bp	Enhancer	Promoter
1	cg05411165	9,894	-0.005	0.001	1.02E-06	1.40E-02	0	9.77E-01	ZNF184 (-25414), HIST1H2BL (+309398)	6	27466334	-	-

Effect: Regression coefficients

FDR: False discovery Rate adjusted P-value, threshold = 0.05

HetISq: Heterogeneity I^2 parameter

HetPVal: Heterogeneity p-value

Enhancer & promoter annotations from Illumina 450k annotation
