# Supplementary material for: Genetically defined elevated homocysteine levels do not result in widespread changes of DNA methylation in leukocytes
Source: PLoS One. 2017 Oct 30;12(10):e0182472. doi: 10.1371/journal.pone.0182472 (PMC5662081; doi:10.1371/journal.pone.0182472)
Supplement: S6 Table — (a) 14 trans-meQTLs of chromosome 6 with FDR<0.05 that are associated with Genetic Risk Score of 18 Hcy-associated variants in a sample size of 9,894, and were 1Mb-5Mb away from the SNP rs548987 of SLC17A3 gene. (b) Conditional analysis for the 14 trans-meQTLs of chromosome 6 with adjustment for the SNP rs548987 of SLC17A3 gene in a subset of 3,786. The CpGs are sorted in base pairs. (PDF) [file pone.0182472.s010.pdf]

S6a Table. 14 *trans*-meQTLs of chromosome 6 with FDR<0.05 that are associated with Genetic Risk Score of 18 Hcy-associated variants in a sample size of 9,894, and were 1Mb-5Mb away from the SNP rs548987 of SLC17A3 gene. The CpGs are sorted in base pairs.

Rank	CpG	Sample Size	Effect	StdErr	Pvalue	FDR	HetISq	HetPVal	Genes Associated	Chr	Bp	Enhancer	Promoter
7	cg18278486	9,894	-0.021	0.004	8.58E-08	5.06E-04	51.2	2.05E-02	ZNF322 (-327572), HIST1H2BJ (+113023)	6	26987575	-	-
10	cg10547057	9,894	-0.010	0.002	4.57E-07	2.29E-03	59.4	4.47E-03	ZNF322 (-327855), HIST1H2BJ (+112740)	6	26987810	-	-
12	cg00903577	9,894	-0.022	0.005	5.51E-07	2.65E-03	25.2	1.96E-01	SCAND3 (-276022), TRIM27 (+60634)	6	28831109	-	TRUE
29	cg03270340	8,095	-0.004	0.001	1.44E-05	4.76E-02	45	5.17E-02	TRIM27 (+587)	6	28891204	-	TRUE
23	cg11747594	9,894	-0.040	0.009	8.52E-06	3.03E-02	32.7	1.29E-01	ZFP57 (-3319)	6	29648225	-	-
25	cg07134666	9,894	-0.051	0.012	1.31E-05	4.49E-02	9.7	3.50E-01	ZFP57 (-3494)	6	29648400	-	-
21	cg16885113	9,894	-0.043	0.010	7.30E-06	2.66E-02	13.1	3.16E-01	ZFP57 (-3601)	6	29648507	-	-
28	cg20228636	9,894	-0.044	0.010	1.43E-05	4.76E-02	16.2	2.85E-01	ZFP57 (-3619)	6	29648525	-	-
27	cg11383134	9,894	-0.045	0.010	1.41E-05	4.72E-02	9.4	3.53E-01	ZFP57 (-3684)	6	29648590	-	-
15	cg13643180	9,894	0.013	0.003	1.69E-06	7.22E-03	2	4.24E-01	HLA-G (-33443), LOC554223 (+1630)	6	29761288	-	TRUE
11	cg15411272	9,894	0.051	0.010	5.15E-07	2.52E-03	9.3	3.54E-01	HLA-A (-15083), HLA-G (+100408)	6	29895187	-	TRUE
8	cg26121931	9,894	0.056	0.011	2.07E-07	1.11E-03	17.7	2.70E-01	HLA-A (-15066), HLA-G (+100425)	6	29895204	-	TRUE
16	cg04628742	9,334	-0.017	0.004	2.36E-06	9.73E-03	0	7.43E-01	HLA-J (-971)	6	29973221	-	-
20	cg13196879	9,894	0.010	0.002	6.78E-06	2.49E-02	34.9	1.11E-01	RNF39 (-27824), TRIM31 (+9415)	6	30071427	-	-

Effect: Regression coefficients

FDR: False discovery Rate adjusted P-value, threshold = 0.05

HetISq: Heterogeneity I^2 parameter

HetPVal: Heterogeneity p-value

Enhancer & promoter annotations from Illumina 450k annotation

S6b Table. Conditional analysis for the 14 *trans*-meQTLs of chromosome 6 with adjustment for the SNP rs548987 of SLC17A3 gene in a subset of 3,786. The CpGs are sorted in base pairs.

			Unconditional analysis (N=3,786)				Conditional analysis (N=3,786) adjusted for rs548987 at chr 6			
Rank	CpG	Sample Size	Effect	StdErr	Pvalue	Bonferroni	Effect	StdErr	Pvalue	Bonferroni
7	cg18278486	3,786	-0.022	0.006	1.33E-04	Yes	-0.006	0.006	2.89E-01	No
10	cg10547057	3,786	-0.014	0.003	1.56E-05	Yes	-0.005	0.003	8.80E-02	No
12	cg00903577	3,786	-0.025	0.006	8.70E-05	Yes	-0.002	0.006	7.94E-01	No
29	cg03270340	3,786	-0.005	0.001	2.05E-04	Yes	-0.002	0.001	1.82E-01	No
23	cg11747594	3,786	-0.029	0.013	2.48E-02	No	0.003	0.014	8.33E-01	No
25	cg07134666	3,786	-0.041	0.019	2.57E-02	No	0.013	0.016	4.12E-01	No
21	cg16885113	3,786	-0.032	0.015	2.78E-02	No	0.007	0.015	6.43E-01	No
28	cg20228636	3,786	-0.035	0.015	1.88E-02	No	0.004	0.015	8.17E-01	No
27	cg11383134	3,786	-0.035	0.016	3.21E-02	No	0.014	0.014	3.34E-01	No
15	cg13643180	3,786	0.010	0.004	1.12E-02	No	0.007	0.004	5.48E-02	No
11	cg15411272	3,786	0.041	0.015	7.13E-03	No	-0.007	0.014	5.92E-01	No
8	cg26121931	3,786	0.050	0.016	2.26E-03	No	0.000	0.016	9.82E-01	No
16	cg04628742	3,786	-0.019	0.006	9.40E-04	Yes	-0.005	0.006	3.72E-01	No
20	cg13196879	3,786	0.012	0.004	1.61E-03	No	0.002	0.003	5.88E-01	No

Effect: Regression coefficients

Bonferroni threshold = 3.57E-03
