# Supplementary material for: Genetically defined elevated homocysteine levels do not result in widespread changes of DNA methylation in leukocytes
Source: PLoS One. 2017 Oct 30;12(10):e0182472. doi: 10.1371/journal.pone.0182472 (PMC5662081; doi:10.1371/journal.pone.0182472)
Supplement: S7 Table — (a) 7 genome-wide trans-meQTLs with FDR<0.05 that are associated with Genetic Risk Score of 18 Hcy-associated variants in a sample size of 9,894 and were a direct trans-meQTL of either SNP rs548987 of SLC17A3 gene or rs154657 SNP of DPEP1 gene. (b) Conditional analysis for the 7 genome-wide trans-meQTLs with adjustment for their respective SNP rs548987 of SLC17A3 gene at chromosome 6 or rs154657 SNP of DPEP1 gene at chromosomes 16 in a subset of 3,786. (PDF) [file pone.0182472.s011.pdf]

S7a Table. 7 genome-wide *trans*-meQTLs with FDR<0.05 that are associated with Genetic Risk Score of 18 Hcy-associated variants in a sample size of 9,894 and were a direct *trans*-meQTL of either SNP rs548987 of SLC17A3 gene or rs154657 SNP of DPEP1 gene.

Rank	CpG	Sample Size	Direct *trans*-meQTL of:	Effect	StdErr	Pvalue	FDR	HetISq	HetPVal	Genes Associated	Chr	Bp	Enhancer	Promoter
19	cg01620082	9,894	rs548987	-0.017	0.004	6.03E-06	2.28E-02	20.5	2.43E-01	ALG1L (-22545), ROPN1B (-9596)	3	125678407	-	-
13	cg06606381	9,894	rs548987	-0.021	0.004	6.45E-07	3.07E-03	30.1	1.51E-01	GALNT9 (-179017), P2RX2 (-110481)	12	133084897	-	-
14	cg17862947	9,334	rs548987	-0.014	0.003	7.56E-07	3.52E-03	47.8	4.48E-02	GALNT9 (-181046), P2RX2 (-108452)	12	133086926	-	-
2	cg24644049	9,894	rs154657	-0.016	0.002	2.24E-20	3.73E-16	69.3	1.77E-04	CDS1 (-32)	4	85504048	-	-
3	cg03958163	9,894	rs154657	-0.010	0.001	7.95E-17	1.03E-12	86.3	1.20E-12	SH3PXD2A (-315)	10	105615502	-	-
22	cg21963436	9,894	rs154657	0.004	0.001	7.65E-06	2.76E-02	57.9	6.16E-03	CDX2 (+2150), PDX1 (+46999)	13	28541142	-	-
9	cg03253314	9,894	rs154657	-0.003	0.001	2.29E-07	1.21E-03	0	6.37E-01	GTPBP3 (-2590), ANO8 (-98)	19	17445711	-	TRUE

Effect: Regression coefficients

FDR: False discovery Rate adjusted P-value, threshold = 0.05

HetISq: Heterogeneity I^2 parameter

HetPVal: Heterogeneity p-value

Enhancer & promoter annotations from Illumina 450k annotation

S7b Table: Conditional analysis for the 7 genome-wide *trans*-meQTLs with adjustment for their respective SNP rs548987 of SLC17A3 gene at chromosome 6 or rs154657 SNP of DPEP1 gene at chromosomes 16 in a subset of 3,786.

				Unconditional analysis (N=3,786)				Conditional analysis (N=3,786) adjusted for rs548987 at chr6				Conditional Analysis (N=3,786) adjusted for rs154657 at chr16			
Rank	CpG	Sample Size	Direct *trans*-meQTL of:	Effect	StdErr	P-value	Bonferroni	Effect	StdErr	P-value	Bonferroni	Effect	StdErr	P-value	Bonferroni
19	cg01620082	3,786	rs548987	-0.012	0.006	3.86E-02	No	0.0083	0.0056	1.37E-01	No	-	-	-	-
13	cg06606381	3,786	rs548987	-0.016	0.007	1.84E-02	No	0.0075	0.0063	2.33E-01	No	-	-	-	-
14	cg17862947	3,786	rs548987	-0.013	0.004	4.02E-03	Yes	0.0012	0.0042	7.70E-01	No	-	-	-	-
2	cg24644049	3,786	rs154657	-0.016	0.003	5.21E-08	Yes	-	-	-	-	-0.0049	0.0031	1.10E-01	No
3	cg03958163	3,786	rs154657	-0.016	0.003	1.08E-10	Yes	-	-	-	-	0.0004	0.0024	8.62E-01	No
22	cg21963436	3,786	rs154657	0.002	0.001	1.82E-01	No	-	-	-	-	0.0007	0.0015	6.47E-01	No
9	cg03253314	3,786	rs154657	-0.003	0.001	2.21E-03	Yes	-	-	-	-	-0.0008	0.0011	4.69E-01	No

Effect: Regression coefficients

Bonferroni threshold = 7.14E-03
