# Supplementary material for: Genetically defined elevated homocysteine levels do not result in widespread changes of DNA methylation in leukocytes
Source: PLoS One. 2017 Oct 30;12(10):e0182472. doi: 10.1371/journal.pone.0182472 (PMC5662081; doi:10.1371/journal.pone.0182472)
Supplement: S8 Table — The CpGs are sorted in chromosomes and base pairs. (PDF) [file pone.0182472.s012.pdf]

S8 Table: 9 genome-wide *trans*-meQTLs with FDR<0.05 that are associated with Genetic Risk Score of 18 Hcy-associated variants in a sample size of 9,894. The CpGs are sorted in chromosomes and base pairs.

Rank	CpG	Sample Size	Effect	StdErr	Pvalue	FDR	HetISq	HetPVal	Genes Associated	Chr	Bp	Enhancer	Promoter
5	cg21302420	6,837	-0.006	0.001	3.98E-12	3.56E-08	64.4	9.77E-03	RAP1A	1	112162376	-	TRUE
6	cg20159777	9,334	-0.005	0.001	6.21E-11	5.16E-07	66.6	1.42E-03	BTG2 (+21911), FMOD (+23714)	1	203296550	-	TRUE
18	cg12805629	6,277	-0.018	0.004	3.15E-06	1.23E-02	0	7.09E-01	MRPL55 (+6698), ARF1 (+19954)	2	11565653	-	-
30	cg08586216	9,894	-0.002	0.001	1.49E-05	4.89E-02	37.6	9.09E-02	TULP1 (-131681), FKBP5 (+44391)	6	35612351	TRUE	-
4	cg03920392	6,837	-0.009	0.001	8.83E-14	8.75E-10	61.9	1.51E-02	ZNF395	8	28243022	-	-
17	cg00620062	9,894	0.004	0.001	2.83E-06	1.12E-02	0	8.69E-01	PTF1A (+6292)	10	23487775	-	-
24	cg00677455	9,334	-0.003	0.001	8.76E-06	3.09E-02	0	6.21E-01	CTDSP2 (-269)	12	58241039	-	TRUE
1	cg13698153	9,733	-0.012	0.001	5.97E-54	5.56E-49	90.8	1.05E-18	VSIG10 (+63)	12	118541722	-	-
26	cg01259782	6,194	0.015	0.004	1.33E-05	4.52E-02	0	7.43E-01	CRYM (+454)	16	21313973	-	-

Effect: Regression coefficients

FDR: False discovery Rate adjusted P-value, threshold = 0.05

HetISq: Heterogeneity I^2 parameter

HetPVal: Heterogeneity p-value

Enhancer & promoter annotations from Illumina 450k annotation
