# Supplementary material for: Genetically defined elevated homocysteine levels do not result in widespread changes of DNA methylation in leukocytes
Source: PLoS One. 2017 Oct 30;12(10):e0182472. doi: 10.1371/journal.pone.0182472 (PMC5662081; doi:10.1371/journal.pone.0182472)
Supplement: S9 Table — (a) MTHFR 677C>T variant associated DMPs at the 3 IGF2/H19 DMR regions at chromosome 11. (b) GRS associated DMPs at the 3 IGF2/H19 DMR regions at chromosome 11. (PDF) [file pone.0182472.s013.pdf]

S9a Table. MTHFR 677C>T variant associated DMPs at the 3 IGF2/H19 DMR regions at chromosome 11.

DMR	CpG	N	Effect	StdErr	Pvalue	Bonferroni	HetISq	Bp
H19-DMR3	cg22259242	++++-+-+--??	0.0006	0.0003	0.089	No	43.4	2021243
DMR2	cg13165070	?????+-+?-++	0.0009	0.0011	0.421	No	10.4	2154113
DMR2	cg11717189	?????---?+++	-0.0003	0.0016	0.835	No	0	2154132
DMR2	cg07096953	-++--+--+--+	0.0004	0.0009	0.643	No	0	2154255
DMR2	cg02613624	-++++----+-+	0.0003	0.0006	0.611	No	21.6	2154386
DMR0	cg00273464	++-+-+-++++-	0.0012	0.0008	0.143	No	0	2170412
DMR0	cg17665927	+-++-++++++-	0.0014	0.0009	0.138	No	0	2170443

Effect: Regression coefficients

Bonferroni threshold = 7.14E-03

HetISq: Heterogeneity I^2 parameter

Enhancer & promoter annotations from Illumina 450k annotation

S9b Table. GRS associated DMPs at the 3 IGF2/H19 DMR regions at chromosome 11.

DMR	CpG	N	Effect	StdErr	Pvalue	Bonferroni	HetISq	Bp
H19-DMR3	cg22259242	++++-+-+--??	-0.0002	0.0012	0.853	No	0	2021243
DMR2	cg13165070	?????+++?+++	0.0015	0.0041	0.719	No	0	2154113
DMR2	cg11717189	?????---?-+-	-0.0097	0.0059	0.099	No	0	2154132
DMR2	cg07096953	-++------+-+	0.0005	0.0033	0.872	No	0	2154255
DMR2	cg02613624	+++-+++--+-+	0.0007	0.0022	0.758	No	4.4	2154386
DMR0	cg00273464	++-+++--+---	0.0001	0.0029	0.965	No	0	2170412
DMR0	cg17665927	+-++++-+----	0.0004	0.0034	0.910	No	0	2170443

Effect: Regression coefficients

Bonferroni threshold = 7.14E-03

HetISq: Heterogeneity I^2 parameter

Enhancer & promoter annotations from Illumina 450k annotation
