# Supplementary material for: Genetically defined elevated homocysteine levels do not result in widespread changes of DNA methylation in leukocytes
Source: PLoS One. 2017 Oct 30;12(10):e0182472. doi: 10.1371/journal.pone.0182472 (PMC5662081; doi:10.1371/journal.pone.0182472)
Supplement: S1 Text — (PDF) [file pone.0182472.s014.pdf]

S1 Text. Description of cohorts

CHARGE cohorts

Rotterdam Study (RS)

The Rotterdam Study (RS) is a large prospective, population-based cohort study aimed at assessing the occurrence of and risk factors for chronic (cardiovascular, endocrine, hepatic, neurological, ophthalmic, psychiatric, dermatological, oncological, and respiratory) diseases in the elderly [1]. The study comprises 14,926 subjects in total, living in the well-defined Ommoord district in the city of Rotterdam in the Netherlands. In 1989, the first cohort, Rotterdam Study-I (RS-I) comprised of 7,983 subjects with age 55 years or above. In 2000, the second cohort, Rotterdam Study-II (RS-II) was included with 3,011 subjects who had reached an age of 55 or over in 2000. In 2006, the third cohort, Rotterdam Study-III (RS-III) was further included with 3,932 subjects with age 45 years and above. Each participant gave an informed consent and the study was approved by the medical ethics committee of the Erasmus University Medical Center, Rotterdam, the Netherlands.

Illumina Infinium Methylation Assay: At the Genetic Laboratory (Department of Internal Medicine, Erasmus University Medical Center, Rotterdam, the Netherlands), the DNA methylation dataset was generated for a subset of 747 individuals of RS-III at baseline. The second DNA methylation dataset was generated in another subset of 864 individuals comprising of individuals at their fifth, third and second visit of RS-I, RS-II and RS3 respectively, between 2009-2013. Genomic DNA was extracted from whole peripheral blood by standardized salting out methods. This was followed by a bisulfite conversion using the Zymo EZ-96 DNA-methylation kit (Zymo Research, Irvine, CA, USA). The genome for each sample was then amplified, fragmented and hybridized to the Infinium Illumina Human Methylation 450k arrays according to the manufacturer's protocol.

Lothian Birth Cohort (LBC)

The Lothian Birth Cohorts of 1921 and 1936 are follow-up studies of the Scottish Mental Surveys of 1932 and 1947. The surveys had, respectively, tested the intelligence of almost every child born in 1921 or 1936 and attending school in Scotland in the month of June in those years. Therefore, tracing, recruiting and re-testing people who had taken part in the Surveys offered a rare opportunity to examine the distribution and causes of cognitive ageing across most of the human life course. The studies described here were initially set up to study determinants of non-

pathological cognitive ageing; i.e. the ageing of cognitive functions largely in the normal range, and not principally dementia or other pathological cognitive disorders.

Illumina Infinium Methylation Assay: DNA methylation was assessed using the Illumina Human Methylation 450k BeadChip from whole blood of consenting participants. Background correction and quality control were performed to remove probes with a low detection rate, low quality based on manual inspection, and samples with a mismatch between genotypes and SNP control probes, and incorrectly predicted sex. Full details are provided in Shah et al. Genome Research 2014.

Cooperative health research in the Region of Augsburg (KORA)

The KORA study is a series of independent population-based epidemiological surveys of participants living in the region of Augsburg, Southern Germany. All survey participants are residents of German nationality identified through the registration office and were examined in 1994/95 (KORA S3) and 1999/2001 (KORA F4). In the KORA S3 and S4 studies 4,856 and 4,261 subjects have been examined implying response rates of 75% and 67%, respectively. 3,006 subjects participated in a 10-year follow-up examination of S3 in 2004/05 (KORA F3), and 3080 of S4 in 2006/2008 (KORA F4). Individuals for genotyping in KORA F3 and KORA F4 were randomly selected. The age range of the participants was 25 to 74 years of recruitment. Informed consent has been given by all participants. The study has been approved by the local ethics committee.

Illumina Infinium Methylation Assay: Genome-wide DNA methylation patterns were analyzed from whole blood in 1799 individuals, using the Infinium HumanMethylation450 BeadChip Array from Illumina.

Framingham Heart Study (FHS)

The FHS began in 1948 with the enrolment of two-thirds of the adult population of Framingham, Massachusetts, including 2873 women aged 28–62 years. In 1971, 5124 offspring of the original cohort members and offspring spouses, called the "Offspring cohort", were enrolled in the Framingham Heart Study, including 2641 women ranging in age from 12 to 60 years.

Illumina Infinium Methylation Assay: Genome-wide DNA methylation assayed from whole blood samples of 2648 Offspring cohort individuals attending the eighth examination cycle (2005-2011), using the Infinium HumanMethylation450 BeadChip Array from Illumina. Genomic DNA was extracted from whole peripheral blood by standardized salting out methods. This was followed by a bisulfite conversion using the Zymo EZ-96 DNA-methylation kit (Zymo Research, Irvine, CA, USA).

The genome for each sample was then amplified, fragmented and hybridized to the Infinium Illumina Human Methylation 450k arrays according to the manufacturer's protocol. Of these 2648 samples, 2408 samples also have genotypic data.

BIOS cohorts

For the five BIOS datasets, RS (RS-II-3/RS-III-2), LLS, LLD, NTR and CODAM, the DNA methylation data was generated and processed identically.

Illumina Infinium Methylation Assay: For the generation of genome-wide DNA methylation data, 500 ng of genomic DNA was bisulfite modified using the EZ DNA Methylation kit (Zymo Research, Irvine, California, USA) and hybridized on Illumina 450k arrays according to the manufacturer's protocols. The original IDAT files were generated by the Illumina iScan BeadChip scanner. We collected methylation data for a total of 3,841 samples. Data was generated by the Human Genotyping facility (HugeF) of ErasmusMC, the Netherlands (www.glimDNA.org).

Leiden Longevity Study (LLS)

The aim of LLS (http://www.molepi.nl/research/longevity), is to identify the mechanisms that contribute to healthy ageing and longevity. 421 families have been enrolled, that are enriched for familial longevity. They comprise 944 nonagenarian siblings, 1671 of their offspring and 744 partners thereof representing the general population. The middle-aged offspring of the nonagenarians display lower incidence of Type 2 Diabetes, Myocardial infarction and Hypertension than the population controls. The recent report shows that the nonagenarians of the LLS carry as many risk alleles for cardiovascular disease, cancer and type 2 diabetes as the younger population controls. This suggests that other genetic and environmental factors present among individuals surviving into old age may counteract the detrimental effects of disease susceptibility alleles.

LifeLines (LLD)

The LifeLines-DEEP (LLD) cohort[12] is a sub-cohort of the LifeLines cohort.[36] LifeLines is a multi-disciplinary prospective population-based cohort study examining the health and health-related behaviours of 167,729 individuals living in the northern parts of The Netherlands using a unique three-generation design. It employs a broad range of investigative procedures assessing the biomedical, socio-demographic, behavioural, physical and psychological factors contributing to health and disease in the general population, with a special focus on multi-morbidity and complex genetics. A subset of 1,500 LifeLines participants also take part in LLD[12]. For these participants,

additional molecular data is generated, allowing for a more thorough investigation of the association between genetic and phenotypic variation.

Netherlands Twin Register (NTR)

The Netherlands Twin Register (NTR) was founded on February 1st 1987 at the Vrije Universiteit in Amsterdam for the purpose of conducting scientific research. A large number of families with young twins are registered with the NTR. These twins are followed from birth in their development. Another important research of the NTR focuses on the health and life styles of adolescents and adults. Approximately 25,000 twins and multiples over 18 years and 62,000 twins and multiples between 0 and 18 years are registered with the NTR. All in all over 175,000 subjects (multiples, parents, siblings, spouses etc.) are registered.

The aim of the NTR is to examine the contribution of hereditary predisposition to personality, growth, development, disease and risk factors for disease. Multiples are not different from singles, but the fact that with the help of twins we can determine to what extent differences between individuals are to be contributed to heredity and environmental factors is unique.

Cohort on Diabetes and Atherosclerosis Maastricht (CODAM)

The CODAM cohort consists of over 500 individuals (301 with normal glucose tolerance; 127 with impaired glucose metabolism, 146 with Type 2 diabetes) who were selected from a large, population-based cohort on the basis of a moderately increased risk to develop type 2 diabetes and/or cardiovascular disease [2]. DNA methylation data has been measured in 188 samples collected from participants at the 1st followup evaluation of CODAM (~7 years from recruitment). A range of demographic, health and lifestyle data, serum biomarkers and clinical measures are available for these participants. Participants are primarily White Dutch, with a mean age of 65 years (range 48-79) and approximately 55% are male. DNA was derived from peripheral whole blood.

Other cohorts

MARseille THrombosis Association Study (MARTHA)

The MARTHA study is a collection of 1,542 patients with VT recruited from the Thrombophilia centre of La Timone hospital (Marseille, France) [3-6]. All subjects had a documented history of VT, were free of chronic conditions, and were free of inherited thrombophilia including: anti-thrombin, protein C and protein S deficiencies and homozygosity for the Factor V Leiden and Factor II

G20210A mutations. For the methylation project, 349 MARTHA patients were randomly selected for DNA methylation analysis [7-10].

Illumina Infinium Methylation Assay: Genomic DNA was isolated from peripheral blood cells using an adaptation of the method proposed by Miller SA, et al. [11] For each sample, 1 µg genomic DNA was bisulphite converted using the Qiagen EpiTect 96 Bisulfite Kit. Then, 200 ng of bisulfite-converted DNA at 50 ng/µl was independently amplified, labeled, and hybridized to Infinium HumanMethylation450 BeadChip microarrays and scanned with default settings using the Illumina iScan. Samples were processed at The Center for Applied Genomics (TCAG, Toronto, Canada).

French-Canadian family study on Factor V Leiden (F5L) thrombophilia (F5L)

Five extended French-Canadian families were ascertained through single probands with idiopathic venous thromboembolism (VT) diagnosed at the Thrombosis Clinic of the Ottawa Hospital, and carrying the FVL mutation. VT cases secondary to cancer as well as rare forms of inherited VT (protein S, protein C, AntiThrombin deficiencies, FVL homozygotes) were excluded. A pedigree was drawn from interviews with each potential probands. The largest families were invited to participate in the study - the family size and willingness to participate being the only criteria for the selection of the families.

Illumina Infinium Methylation Assay DNA was extracted from peripheral blood using a salting out procedure adapted from [11]. Bisulfite conversion and DNA methylation measurements were performed at The Center for Applied Genomics, Toronto, Canada in 227individuals as described elsewhere [8]. Bisulfite conversion was performed on 1 µg genomic DNA for each sample using the Qiagen EpiTect 96 Bisulfite Kit and 200 ng of bisulfite-converted DNA at 50 ng/µl was independently amplified, labeled and hybridized to Infinium HumanMethylation450 BeadChip microarrays.

References

1. Hofman A, Brusselle GG, Darwish Murad S, van Duijn CM, Franco OH, Goedegebure A, et al. The Rotterdam Study: 2016 objectives and design update. European Journal of Epidemiology 2015;30:661-708.
2. van Greevenbroek MM, Jacobs M, van der Kallen CJ, Blaak EE, Jansen EH, Schalkwijk CG, et al. Human plasma complement C3 is independently associated with coronary heart disease, but only in heavy smokers (the CODAM study). Int J Cardiol 2012;154:158-62.
3. Antoni G, Morange PE, Luo Y, Saut N, Burgos G, Heath S, et al. A multi-stage multi-design strategy provides strong evidence that the BAI3 locus is associated with early-onset venous thromboembolism. J Thromb Haemost 2010;8:2671-9.
4. Antoni G, Oudot-Mellakh T, Dimitromanolakis A, Germain M, Cohen W, Wells P, et al. Combined analysis of three genome-wide association studies on vWF and FVIII plasma levels. BMC Med Genet 2011;12:102.
5. Huang J, Sabater-Lleal M, Asselbergs FW, Tregouet D, Shin SY, Ding J, et al. Genome-wide association study for circulating levels of PAI-1 provides novel insights into its regulation. Blood 2012;120:4873-81.
6. Oudot-Mellakh T, Cohen W, Germain M, Saut N, Kallel C, Zelenika D, et al. Genome wide association study for plasma levels of natural anticoagulant inhibitors and protein C anticoagulant pathway: the MARTHA project. Br J Haematol 2012;157:230-9.
7. Dick KJ, Nelson CP, Tsaprouni L, Sandling JK, Aissi D, Wahl S, et al. DNA methylation and body-mass index: a genome-wide analysis. Lancet 2014;383:1990-8.
8. Gagnon F, Aissi D, Carrie A, Morange PE, Tregouet DA. Robust validation of methylation levels association at CPT1A locus with lipid plasma levels. J Lipid Res 2014;55:1189-91.
9. Aissi D, Dennis J, Ladouceur M, Truong V, Zwingerman N, Rocanin-Arjo A, et al. Genome-wide investigation of DNA methylation marks associated with FV Leiden mutation. PLoS One 2014;9:e108087.
10. Rocanin-Arjo A, Dennis J, Suchon P, Aissi D, Truong V, Tregouet DA, et al. Thrombin generation potential and whole-blood DNA methylation. Thromb Res 2015;135:561-4.
11. Miller SA, Dykes DD, Polesky HF. A simple salting out procedure for extracting DNA from human nucleated cells. Nucleic Acids Res 1988;16:1215.
