# Supplementary material for: Genetically defined elevated homocysteine levels do not result in widespread changes of DNA methylation in leukocytes
Source: PLoS One. 2017 Oct 30;12(10):e0182472. doi: 10.1371/journal.pone.0182472 (PMC5662081; doi:10.1371/journal.pone.0182472)
Supplement: S2 File — (PDF) [file pone.0182472.s016.pdf]

Members of the CHARGE Consortium

NAME	EMAIL	DEPARTMENT	INSTITUTION/UNIVERSITY	CITY	COUNTRY
Myriam Fornage (Group Leader)	myriam.fornage@uth.tmc.edu	Institute of Molecular Medicine	The University of Texas Health Science Center at Houston	Houston	USA
Abbas Dehghan	a.dehghan@imperial.ac.uk	Department of Epidemiology and Biostatistics	Imperial College London	London	UK
Adrienne Tin	atin1@jhu.edu	Department of Epidemiology	Johns Hopkins Bloomberg School of Public Health	Baltimore	USA
Alexander Teumer	ateumer@uni-greifswald.de	Institute of Community Medicine	University of Greifswald	Greifswald	Germany
Ali Shojaie	ashojaie@uw.edu	Department of Biostatistics	University of Washington	Seattle	USA
Allan Just	acjust@hsph.harvard.edu	Department of Environmental Health	Harvard T.H. Chan School of Public Health	Boston	USA
Andre Uitterlinden	a.g.uitterlinden@erasmusmc.nl	Department of Internal Medicine	Erasmus University Medical Center	Rotterdam	Netherlands
Anja Kretschmer	anja.kretschmer@helmholtz-muenchen.de	Institute of Epidemiology II	Helmholtz Zentrum München	Munich	Germany
Anne Justice	justanne@email.unc.edu	Department of Epidemiology	University of North Carolina	Chapel Hill	USA
Audrey Chu	audrey.chu@nih.gov	Division of Intramural Research	National Heart, Lung and Blood Institute	Bethesda	USA
Baccarelli, Andrea	andrea.baccarelli@columbia.edu	Environmental Health Sciences	Columbia University	New York	USA
Bertha Hidalgo	bhidalgo@uab.edu	Department of Epidemiology	UAB School of Public Health	Birmingham	USA
Bonnie Joubert	bonnie.joubert@nih.gov	Population Health Branch	National Institute of Environmental Health Sciences	Durham	USA
Brenton Swenson	brenton.swenson@gmail.com	Cardiovascular Health Research Unit	University of Washington	Seattle	USA

Bressler, Jan	Jan.Bressler@uth.tmc.edu	Epidemiology,<br>Human Genetics & Environmental Sciences	The University of Texas Health Science Center at Houston	Houston	USA
Brian Chen	brian.chen@nih.gov	-	National Institute on Aging	Bethesda	USA
Bruce Psaty	psaty@u.washington.edu	Department of Biostatistics	University of Washington	Seattle	USA
Carola Marzi	carola.marzi@helmholtz-muenchen.de	Institute of Molecular Epidemiology	Helmholtz Zentrum München	Munich	Germany
Caroline Relton	Caroline.Relton@bristol.ac.uk	School of Social and Community Medicine	University of Bristol	Bristol	UK
Cathy Elks	Cathy.Elks@mrc-epid.cam.ac.uk	MRC Epidemiology Unit	University of Cambridge	Cambridge	UK
Cavin Ward-Caviness	cavin.ward-caviness@helmholtz-muenchen.de	Institute of Epidemiology II	Helmholtz Zentrum München	Munich	Germany
christian gieger	christian.gieger@helmholtz-muenchen.de	Institute of Molecular Epidemiology	Helmholtz Zentrum München	Munich	Germany
Christian Herder	Christian.Herder@DDZ.uni-duesseldorf.de	Institute of Clinical Diabetology,<br>German Diabetes Center	Heinrich Heine University Düsseldorf	Düsseldorf	Germany
Chunyu Liu	chunyu.liu@nih.gov	Division of Intramural Research	National Heart, Lung and Blood Institute	Bethesda	USA
Paul Courchesne	courchesnepl@nhlbi.nih.gov	Division of Intramural Research	National Heart, Lung and Blood Institute	Bethesda	USA
Dana Hernandez	hernand@mail.nih.gov	-	National Institute on Aging	Bethesda	USA
David Melzer	D.Melzer@exeter.ac.uk	Institute of Public Health	University of Cambridge	Cambridge	UK
David Siscovick	dsiscovick@nyam.org	Institute for Urban Health	The New York Academy of Medicine	New York	USA
Donna Arnett	arnett@uab.edu	Department of Epidemiology	UAB School of Public Health	Birmingham	USA

Doug Kiel	kiel@hsl.harvard.edu	Musculoskeletal Research Center	Institute for Aging Research,<br>Hebrew SeniorLife	Boston	USA
Elena Carnero	elena.carnero_montoro@kcl.ac.uk	Division of Genetics and Molecular Medicine	King's College London	London	UK
Elena Colicino	ecolicin@hsph.harvard.edu	Department of Environmental Health	Harvard T.H. Chan School of Public Health	Boston	USA
Ellen Demerath	ewd@umn.edu	Division of Epidemiology and Community Health	University of Minnesota	Minneapolis	USA
Eric Whitsel	eric_whitsel@med.unc.edu	Department of Epidemiology	University of North Carolina	Chapel Hill	USA
Erin Ware	ebakshis@umich.edu	Survey Research Center,<br>Institute for Social Research	University of Michigan	Ann Arbor	USA
Eva Reischl	eva.reischl@helmholtz-muenchen.de	Institute of Molecular Epidemiology	Helmholtz Zentrum München	Munich	Germany
Gail Davies	gail.davies@ed.ac.uk	School of Philosophy,<br>Psychology and Language Sciences	The University of Edinburgh	Edinburgh	UK
Golareh Agha	gagha@hsph.harvard.edu	Department of Environmental Health	Harvard T.H. Chan<br>School of Public Health	Boston	USA
Megan L Grove-Gaona	Megan.L.Grove@uth.tmc.edu	Human Genetic Center	University of Texas Health Science Center	Houston	USA
Gudny Eiriksdottir	gudny@hjarta.is	Molecular Genetics Laboratory	Icelandic Heart Association	Kopavogur	Iceland
Elizabeth Hibler	elizabeth.a.hibler@vanderbilt.edu	Medicine	Vanderbilt University	Nashville	USA
Hortensia Moreno	hortensiamor@gmail.com	-	National Institute of Public Health	Cuernavaca	Mexico
Hugo Aparicio	Hugo.Aparicio@bmc.org	University School of Medicine	Boston Medical Center	Boston	USA
Ian Deary	ian.deary@ed.ac.uk	School of Philosophy,<br>Psychology and Language Sciences	The University of Edinburgh	Edinburgh	UK

Ida Chen	ichen@labiomed.org	Department of Pediatrics	Harbor-UCLA Medical Center	Torrance	USA
Isabelle Romieu	iromieu@gmail.com	Environmental Health Department	National Institute of Public Health	Cuernavaca	Mexico
Ivana Nedeljkovic	i.nedeljkovic@erasmusmc.nl	Department of Epidemiology	Erasmus University Medical Center	Rotterdam	Netherlands
James Floyd	jfloyd@uw.edu	Cardiovascular Health Research Unit	University of Washington	Seattle	USA
Jari Lahti	jari.lahti@helsinki.fi	Helsinki Collegium for Advanced Studies	University of Helsinki	Helsinki	Finland
Jennifer Brody	jeco@u.washington.edu	Cardiovascular Health Research Unit	University of Washington	Seattle	USA
Jeff O'Connell	joconnel@medicine.umaryland.edu	School of Medicine	University of Maryland	Baltimore	USA
Jen Smith	smjenn@umich.edu	School of Public Health	University of Michigan	Ann Arbor	USA
Jerry Rotter	Jrotter@labiomed.org	Institute for Translational Genomics and Population Sciences	Harbor-UCLA Medical Center	Torrance	USA
Jiantao Ma	jiantao.ma@nih.gov	Division of Intramural Research	National Heart, Lung and Blood Institute	Bethesda	USA
Jim Pankow	panko001@umn.edu	Division of Epidemiology and Community Health	University of Minnesota	Minneapolis	USA
Joanne Murabito	murabito@bu.edu	School of Medicine	Boston University	Boston	USA
Jordana Bell	jordana.bell@kcl.ac.uk	Department of Twin Research & Genetic Epidemiology	King's College London	London	UK
Jordi Jimenez	jjimenez@imim.es	The Spanish Stroke Genetics Consortium	GeneStroke	Barcelona	Spain
Joyce van meurs	j.vanmeurs@erasmusmc.nl	Department of Internal Medicine	Erasmus University Medical Center	Rotterdam	Netherlands

Juan castillo_fernandez	juan.castillo_fernandez@kcl.ac.uk	Department of Twin Research & Genetic Epidemiology	King's College London	London	UK
Karen Conneely	kconnee@emory.edu	Department of Human Genetics	Emory University	Atlanta	USA
Katharina Schramm	katharina.schramm@helmholtz-muenchen.de	Institute of Genetic Epidemiology	Helmholtz Zentrum München	Munich	Germany
Kathy Lunetta	klunetta@bu.edu	School of Public Health	Boston University	Boston	USA
Ken Ong	Ken.Ong@mrc-epid.cam.ac.uk	MRC Epidemiology Unit	University of Cambridge School of Clinical Medicine	Cambridge	UK
Kim Braun	k.braun@erasmusmc.nl	Department of Epidemiology	Erasmus University Medical Center	Rotterdam	Netherlands
Layal Chaker	l.chaker@erasmusmc.nl	Department of Epidemiology	Erasmus University Medical Center	Rotterdam	Netherlands
Lei Liu	lei.liu@northwestern.edu	Feinberg School of Medicine	Northwestern University	Chicago	USA
Lekki Frazier-Wood	LekkiWood@Gmail.com	Department of Biostatistics	University of Alabama	Birmingham	UK
Daniel Levy	levyd@nhlbi.nih.gov	Division of Intramural Research	National Heart, Lung and Blood Institute	Bethesda	USA
Lifang Hou	l-hou@northwestern.edu	Feinberg School of Medicine	Northwestern University	Chicago	USA
liliane pfeiffer	liliane.pfeiffer@helmholtz-muenchen.de	Institute of Molecular Epidemiology	Helmholtz Zentrum München	Munich	Germany
Linda Broer	l.broer@erasmusmc.nl	Department of Internal Medicine	Erasmus University Medical Center	Rotterdam	Netherlands
Lindsay Reynolds	lireynol@wakehealth.edu	Department of Epidemiology and Prevention, Division of Public Health Sciences	Wake Forest School of Medicine	Winston-Salem	USA
Liming Liang	lliang@hsph.harvard.edu	Department of Epidemiology	Harvard T.H. Chan School of Public Health	Boston	USA

Luke Pilling	L.Pilling@exeter.ac.uk	Epidemiology and Public Health group	Exeter Medical School	Exeter	UK
Marco Medici	m.medici@erasmusmc.nl	Rotterdam Thyroid Center,<br>Department of Internal Medicine	Erasmus University Medical Center	Rotterdam	Netherlands
Maria Argos	argos@uic.edu	School of Public Health	University of Illinois	Chicago	USA
Marilyn C Cornelis	marilyn.cornelis@northwestern.edu	Feinberg School of Medicine	Northwestern University	Chicago	USA
Mark Omid Goodarzi	mark.goodarzi@cshs.org	Division of Endocrinology	UCLA Clinical and<br>Translational Science Institute	Los Angeles	USA
May Montasser	mmontass@medicine.umaryland.edu	School of Medicine	University of Maryland	Baltimore	USA
Melanie Waldenberger	waldenberger@helmholtz-muenchen.de	Institute of Molecular Epidemiology	Helmholtz Zentrum München	Munich	Germany
Michael Mendelson	Michael.Mendelson@cardio.chboston.org	Department of Cardiology	Boston Children's Hospital	Boston	USA
Mina Jhun	minajhun@umich.edu	Department of Epidemiology	University of Michigan<br>School of Public Health	Ann Arbor	USA
Nancy Heard-Costa	nheard@bu.edu	School of Medicine	Boston University	Boston	USA
Nona Sotoodehnia	nsotoo@u.washington.edu	Cardiovascular Health Research Unit	University of Washington	Seattle	USA
Kari North	kari_north@unc.edu	Department of Epidemiology	University of North Carolina	Chapel Hill	USA
Olivera Jovanova	o.jovanova@erasmusmc.nl	Department of Epidemiology	Erasmus University Medical Center	Rotterdam	USA
Pooja Mandaviya	p.mandaviya@erasmusmc.nl	Department of Internal Medicine	Erasmus University Medical Center	Rotterdam	Netherlands
Paul Yousefi	Paul.yousefi@bristol.ac.uk	School of Social and Community Medicine	University of Bristol	Bristol	UK

Pei-Chien Tsai	pei-chien.tsai@kcl.ac.uk	Twin Research Unit	King's College London	London	UK
Rachel Hennein	rachel.hennein@nih.gov	Framingham Heart Study	National Institutes of Health	Bethesda	USA
Rahul Gondalia	rahgonda@unc.edu	Department of Epidemiology	University of North Carolina	Chapel Hill	USA
Rebecca Richmond	Rebecca.richmond@bristol.ac.uk	Integrative Epidemiology Unit,<br>School of Social and Community Medicine	University of Bristol	Bristol	UK
Elosua Llanos	Relosua@imim.es	Nutrition and Genomics Laboratory	Tufts University	Boston	USA
Riccardo Marioni	Riccardo.Marioni@ed.ac.uk	Centre for Genomic and Experimental Medicine, MRC Institute of Genetics and Molecular Medicine	University of Edinburgh	Edinburgh	UK
Richard, Melissa A	Melissa.A.Lee@uth.tmc.edu	Health Science Center	University of Texas	Houston	USA
Roby Joehanes	robyjoehanes@hsl.harvard.edu	Department of Medicine	Institute for Aging Research,<br>Hebrew SeniorLife	Boston	USA
Rozenn Lemaitre	rozenl@u.washington.edu	Department of Epidemiology	University of Washington	Seattle	USA
Ryan Irvin	irvinr@uab.edu	Department of Epidemiology	UAB School of Public Health	Birmingham	USA
S Ligthart	s.ligthart@erasmusmc.nl	Department of Epidemiology	Erasmus University Medical Center	Rotterdam	Netherlands
Sergi Sayols-Baixeras	ssayols@imim.es	Cardiovascular risk and nutrition	Instituto Hospital del Mar de Investigaciones Médicas	Barcelona	Spain
Sharon Kardia	skardia@umich.edu	Public Health Genetics Program,<br>Life Sciences and Society Program	University of Michigan	Ann Arbor	USA
Sina Gharib	sagharib@u.washington.edu	Division of Pulmonary and Critical Care Medicine	University of Washington	Seattle	USA
Sonja Zeilinger	sonja.zeilinger@helmholtz-muenchen.de	Institute of Molecular Epidemiology	Helmholtz Zentrum München	Munich	Germany

Marla Spires	mspir2@uky.edu	Dean's Office	University of Kentucky<br>College of Public Health	Lexington	USA
Stella Aslibekyan	saslibek@uab.edu	Department of Epidemiology	University of Alabama	Birmingham	UK
Stephanie London	london2@niehs.nih.gov	Epidemiology Branch	National Institute of Environmental Health Sciences	Morrisville	USA
Su, Shaoyong	SSU@gru.edu	Georgia Prevention Institute,<br>Medical College of Georgia	Georgia Regents University	Augusta	USA
Sudha Seshadri	suseshad@bu.edu	Alzheimer's Disease Center	Boston University	Boston	USA
Tao Xu	tao.xu@helmholtz-muenchen.de	Institute of Molecular Epidemiology	Helmholtz Zentrum München	Munich	Germany
Tianxiao Huan	tianxiao.huan@nih.gov	Division of Intramural Research	National Heart, Lung and Blood Institute	Bethesda	USA
Tim Assimes	tassimes@stanford.edu	Department of Medicine - Cardiovascular Medicine	Stanford University	Stanford	USA
Tim Spector	tim.spector@kcl.ac.uk	Department of Twin Research &<br>Genetic Epidemiology	King's College London	London	UK
Tiphaine Martin	tiphaine.martin@kcl.ac.uk	Department of Twin Research and<br>Genetic Epidemiology	King's College London	London	UK
Toshiko Tanaka	tanakato@mail.nih.gov	Longitudinal Studies Section	National Institute on Aging	Bethesda	USA
Trudy Voortman	trudy.voortman@erasmusmc.nl	Department of Epidemiology	Erasmus University Medical Center	Rotterdam	Netherlands
Wei Zhang	weizhang.chicago@gmail.com	Department of Pediatrics	University of Illinois	Chicago	USA
Wei Zhao	zhaowei@umich.edu	Department of Epidemiology	University of Michigan	Ann Arbor	USA
Weihua Guan	wguan@umn.edu	Division of Biostatistics	University of Minnesota	Minneapolis	USA

Yi Hsang Hsu	YiHsiangHsu@hsl.harvard.edu	Gerontology Division	Harvard Medical School	Boston	USA
Yinan Zheng	YinanZheng2011@u.northwestern.edu	Institute for Public Health and Medicine	Northwestern University Feinberg School of Medicine	Chicago	USA
Yongmei Liu	yoliu@wakehealth.edu	Department of Epidemiology & Prevention	Wake Forest Baptist Medical Center	Winston-Salem	USA
Yun Li	yun_li@med.unc.edu	Department of Biostatistics, Department of Genetics	University of North Carolina	Chapel Hill	USA
Zdenka Pausova	zdenka.pausova@sickkids.ca	Hospital for Sick Children	University of Toronto	Toronto	Canada
Zenobia Moore	ann.moore@nih.gov	Division of Lung Diseases	National Heart, Lung and Blood Institute	Bethesda	USA
